# Effects of In-Person Assistance vs Personalized Written Resources About Social Services on Household Social Risks and Child and Caregiver Health: A Randomized Clinical Trial

**DOI:** 10.1001/jamanetworkopen.2020.0701

**Published:** 2020-03-10

**Authors:** Laura M. Gottlieb, Nancy E. Adler, Holly Wing, Denisse Velazquez, Victoria Keeton, Abigail Romero, Maricarmen Hernandez, Andrea Munoz Vera, Elizabeth Urrutia Caceres, Catherine Arevalo, Philip Herrera, Mara Bernal Suarez, Danielle Hessler

**Affiliations:** 1 Social Interventions Research and Evaluation Network, Department of Family and Community Medicine, University of California, San Francisco; 2 School of Nursing, Department of Family Health Care Nursing, University of California, San Francisco; 3 San Francisco State University, San Francisco, California; 4 Department of Urology, University of California, San Francisco; 5 Department of Family and Community Medicine, University of California, San Francisco; 6 Zuckerberg San Francisco General Hospital and Trauma Center, University of California, San Francisco

## Abstract

**Importance:**

Social and economic contexts shape children’s short- and long-term health. Efforts to address contextual risk factors are increasingly incorporated into pediatric health care.

**Objective:**

To compare the effectiveness of 2 social risk–related interventions.

**Design, Setting, and Participants:**

This randomized clinical trial included English- and/or Spanish-speaking caregiver-child dyads recruited from a pediatric urgent care clinic nested in a large, urban, safety-net hospital. Study recruitment, enrollment, and follow-up were conducted from July 18, 2016, to March 8, 2019. Data analysis was conducted from January 1, 2019, to January 20, 2020.

**Interventions:**

Following standardized social risk assessment, caregivers were randomly assigned to receive either written information regarding relevant government and community social services resources or comparable written information plus in-person assistance and follow-up focused on service access.

**Main Outcomes and Measures:**

Caregiver-reported number of social risk factors and child health 6 months after enrollment.

**Results:**

Among 611 caregiver-child dyads enrolled in the study, 302 dyads were randomized to the written resources group and 309 dyads were randomized to the written resources plus in-person assistance group. The mean (SD) age of children was 6.1 (5.0) years; 483 children (79.1%) were Hispanic; and 315 children (51.6%) were girls. There were no significant differences between groups in the effects of the interventions. In post hoc secondary analyses, the number of reported social risks decreased from baseline to 6-month follow-up in both groups: caregivers who received written resources alone reported a mean (SE) of 1.28 (0.19) fewer risks at follow-up, while those receiving written resources plus in-person assistance reported 1.74 (0.21) fewer risks at follow-up (both *P* < .001). In both groups, there were small but statistically significant improvements from baseline to follow-up in child health (mean [SE] change: written resources, 0.37 [0.07]; written resources plus in-person assistance, 0.24 [0.07]; both *P* < .001).

**Conclusions and Relevance:**

This randomized clinical trial compared 2 approaches to addressing social risks in a pediatric urgent care setting and found no statistically significant differences in the social risk and child and caregiver health effects of providing written resources at the point of care with vs without in-person longitudinal navigation services. Caregivers in both groups reported fewer social risks and improved child and caregiver health 6 months after the intervention. These findings deepen understanding of effective doses of social risk–related interventions.

**Trial Registration:**

ClinicalTrials.gov Identifier: NCT02746393

## Key Points

**Question:**

Did in-person, longitudinal social services navigation assistance result in greater reduction in social risk factors and better health for children and caregivers than a less intensive approach?

**Findings:**

In this randomized clinical trial including 611 caregiver-child dyads, there were no statistically significant differences between the groups in the effectiveness of the interventions. In post hoc secondary analyses of both groups, there were significant improvements in social risk factors and in child and caregiver health at 6-month follow-up.

**Meaning:**

These findings suggest that delivery of curated, individualized written resources with or without longitudinal, in-person navigation services about social services in pediatric urgent care settings can affect family circumstances and child and caregiver health.

## Teaser

This randomized clinical trial examined the effect of in-person social services navigation assistance vs a written information sheet on caregiver-reported social risk factors and child and caregiver health.

## Introduction

Childhood social and economic adversity, such as food insecurity and housing instability, is associated with significant short- and long-term health risks, including higher likelihood of childhood behavioral problems, cognitive delay, unhealthy weight, and asthma, as well as adult obesity, diabetes, and cardiovascular disorders.^1,2,3,4,5,6,7,8,9,10,11,12,13,14,15^ Moreover, cumulative exposure to social and economic risk factors has been associated with worse health outcomes independent of the effects of individual risks.^8,16^

In response to consistent and compelling evidence linking social and economic risks to health and well-being, multiple medical professional organizations have endorsed increased attention to social risk factors in the context of clinical care.^17,18,19^ Although pediatricians have long recognized the influence of social context on health outcomes,^20^ recent recommendations have accelerated social risk screening and social risk–related interventions in pediatric clinical settings.^21,22,23,24,25,26,27,28,29,30,31,32,33,34,35,36,37,38,39,40,41,42,43,44,45,46^

Despite considerable diversity in existing pediatric clinical activities aimed at identifying and reducing social risk, little research has rigorously examined program effects or assessed the feasibility of scaling programs across pediatrics clinical settings.^47^ In this study, we compare the effectiveness of 2 social risk–related interventions that differ in intensity and potential scalability: in-person social services navigation assistance offered for up to 3 months by a trained volunteer vs standardized, written information about community and government social services resources. Based on previous research,^23,25,48^ we hypothesized that ongoing, in-person assistance would be more likely to reduce social risks and improve both child and caregiver health outcomes than would 1-time receipt of individualized information about relevant social resources.

## Methods

This 2-group randomized clinical trial was approved by the institutional review board of the University of California, San Francisco. All adult caregivers completed written informed consent, and assent for participation was obtained from children 7 years or older. The complete study protocol is provided in Supplement 1. This study is reported following the Consolidated Standards of Reporting Trials (CONSORT) reporting guideline.

### Study Setting and Participant Eligibility

Study recruitment, enrollment, and follow-up were conducted from July 18, 2016, to March 8, 2019, in the pediatric urgent care clinic at an urban safety-net hospital serving primarily low-income, racially and ethnically diverse populations. Eligible participants were caregiver-child dyads with English- or Spanish-speaking caregivers 18 years or older who were familiar with the child’s household environment and residing in the county of enrollment. Eligible children were aged 0 to 17 years. Only 1 child and 1 caregiver per household were enrolled. If 2 caregivers presented with the child, caregivers self-selected 1 to participate in the study. Families enrolled in a similar primary care–based hospital social services navigation initiative in the 6 months prior or subsequent to recruitment, children in foster care, or those being seen for physical abuse evaluations were excluded (Figure).

**Figure.**
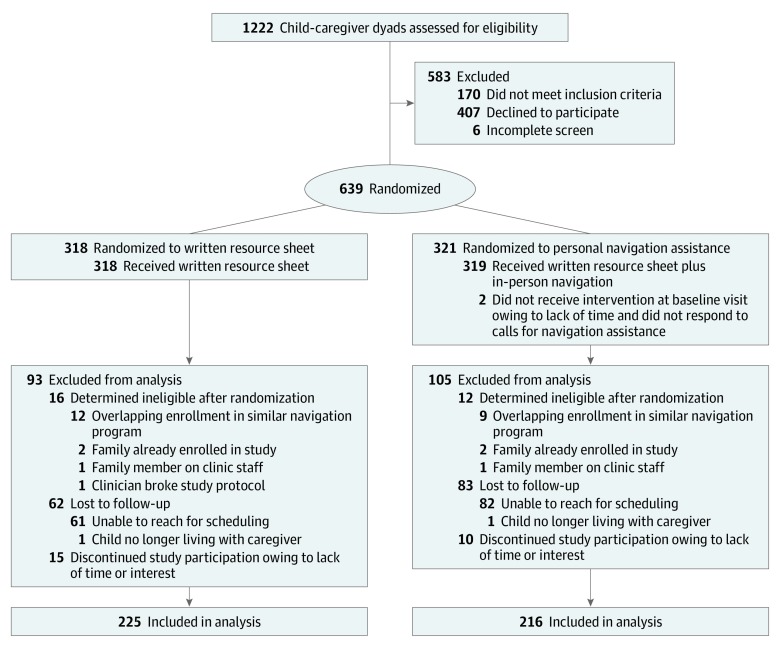
CONSORT Flow Diagram

### Study Procedures, Randomization, and Follow-up

Trained research assistants screened English- or Spanish-speaking families presenting in pediatric urgent care weekdays from 8:30 am to 5:00 pm. Consenting dyads completed all baseline study activities in the clinical examination room during or directly after the child’s medical visit. Research assistants administered baseline demographic characteristics and child health surveys on a tablet computer using Research Electronic Data Capture tools.^49^ Caregivers and children were then given the option of self-completing or working with a research assistant to complete additional child and caregiver health questions. Biomarker samples also were collected from consenting families, although results from those analyses are not presented here. Participating dyads received a $50 gift card after completing baseline study activities.

After informed consent forms were completed and baseline data were collected, the research assistant left the room, and a patient navigator administered a brief social risk survey. The navigator then randomly assigned the participating dyad into 1 of 2 study groups using an automated computer-generated algorithm; randomization occurred within blocks (block size, 10 dyads). Navigators were trained to provide social service navigation services and were periodically observed by supervising senior clinical research associates to ensure they followed study protocols. They were not involved with follow-up outcomes assessment.

#### Written Resources Intervention

Dyads randomized to receive written resources were given information about relevant government, hospital, and community social services. Prewritten informational handouts were populated with data on local resources based on the accumulated knowledge of navigators working in a similar hospital-based linkage program. Navigators individualized the resource sheets by highlighting resources most closely related to the top 3 priority social risk factors endorsed by the caregiver and by providing names of contacts at relevant organizations when available.

#### In-Person Assistance Intervention

Families randomized to in-person navigation services either received assistance immediately after randomization or were offered a telephone or in-person follow-up appointment at a different time. As in the written resources group, during these visits navigators provided written information about resources related to the caregiver’s prioritized social risk factors. Additionally, navigators helped to schedule appointments, complete forms, or provide other social services–related counseling and assistance. Information about protocols for providing written resources and additional supports is published elsewhere.^50^ Following the initial visit, caregivers in the in-person assistance group were contacted by navigators by telephone, text message, or email every 2 weeks for 3 months until identified needs were met or caregivers declined further assistance, whichever came first.

Participating caregivers in both groups were contacted by telephone (and text message, email, and/or letters when telephone calls were unsuccessful) to schedule follow-up interviews 6 months after enrollment. Research assistants, still blinded to study group assignment, administered follow-up surveys using tablet computers. Follow-up interviews were conducted primarily in person in the pediatric urgent care clinic or interview rooms located in the same clinical building. If participants were unable to return to the study site (eg, moved out of the area or had work-related barriers), research assistants completed surveys during home visits or administered surveys by telephone. Participating dyads again received $50 gift cards in exchange for time spent on study activities.

### Measures

#### Demographic Characteristics and Social Risk Factors

Caregivers reported caregiver and/or child age, gender, race, Hispanic/Latino ethnicity, preferred language, family income, caregiver education level, and caregiver-child relationship. At baseline and 6-month follow-up, caregivers answered yes or no questions about their child and household’s social situation. The 18-item social risk screening questionnaire was based on surveys used in earlier studies^25,26^ and included items on housing stability and habitability, food and income security, child care and transportation needs, employment, legal concerns, medical insurance, and other public benefits enrollment (eAppendix in Supplement 2). Additional questions included whether the child and caregiver were connected to primary care, concerns about the child’s experiences of bullying, and adult household members’ mental health.

#### Child Health 

At baseline and 6-month follow-up, child quality of life was assessed with the shortened version of the Pediatric Quality of Life Inventory (PedsQL) 4.0 Generic Core Scales developed for children ages 2 to 18 years,^51^ the PedsQL 4.0 short form 15,^52^ or the PedsQL Infant Scales developed for children ages 1 to 24 months.^53^ The PedsQL uses parent proxy reports to assess child functioning across the following domains: physical functioning, emotional functioning, social functioning, and cognitive or school functioning.^54,55^ Each item is scored on a 5-point Likert scale ranging from 0, indicating never a problem, to 4, almost always a problem, and converted to a 100-point scale, with higher scores indicating higher quality of life. Child global health was assessed by caregiver report using a single item from the 2016 to 2017 National Survey of Children’s Health. This item is associated with health services use and other measures of child mental and physical health (range, 1-5, with higher score indicating better health).^56,57,58^ Caregivers also reported the number of days in the past 6 months the child had missed school, camp, or other activities owing to illness.^59^

#### Caregiver Physical and Mental Health

At baseline and 6-month follow-up, caregivers’ current and recent physical and mental health status was assessed using a single global health item from the Patient-Reported Outcomes Measurement System Global short form version 1.1 (range, 1-5, with higher score indicating better health), which has been sensitive to change in other intervention studies.^60^ Caregivers also completed the 4-item Perceived Stress Scale (range, 0-16, with higher score indicating more perceived stress)^61,62^ and the 8-item Patient Health Questionnaire (range, 0-24, with higher score indicating greater symptoms of depression).^63^

### Sample Size

The initial target sample size was 724 dyads, selected as a conservative sample size to allow for 80% power to detect effects similar to our previous work,^25^ assuming 30% participant attrition. Owing to limitations in study funding, the study concluded with a sample of 611 dyads. The sample gave us approximately 80% power to detect small to medium standard effect sizes of 0.28 or greater between groups and effect sizes of 0.20 or greater to detect changes from preintervention to postintervention within each group.

### Statistical Analysis

Baseline participant characteristics were compared between study groups and tested for significance using χ^2^ or *t* tests and appropriate nonparametric tests for nonnormally distributed continuous variables. The cumulative number of social risks and health outcomes were compared by group assignment using generalized linear models with a normal distribution with identity link for continuous outcomes, Poisson distribution with log link for count outcomes, and binomial distribution with logit link for binary outcomes. Hypothesis tests were 2-sided with *P* < .05 considered statistically significant. Unadjusted differences between groups are presented, and adjustment models built on each other, including (1) adjusted with baseline outcomes entered as covariates and (2) child and caregiver demographic factors (ie, age, gender, and race/ethnicity) entered as covariates. In post hoc secondary analyses, we examined change in outcomes from baseline to follow-up within each group. Statistical analyses were conducted using SPSS statistical software version 25 (IBM Corp). Data were analyzed from January 1, 2019, to January 20, 2020.

## Results

### Sample Characteristics

Of the 1222 families approached, 611 caregiver-child dyads were eligible and agreed to participate (Figure). Child mean (SD) age was 6.1 (5.0) years; 438 children (79.1%) were Hispanic; and 315 children (51.6%) were girls (Table 1). In total, 302 dyads were randomized to the written resources group and 309 dyads to the in-person assistance group. Attrition at follow-up was 27.8%, comparable to similar studies in safety-net settings,^22,64,65^ with no differences between groups. A total of 225 dyads in the written resource sheet group and 216 dyads in the in-person assistance group were included in 6-month follow-up (Figure). There were no statistically significant differences between participants with follow-up data vs participants who dropped out. Using multiple imputation models to estimate missing follow-up data, we also found no significant differences in the number of social risk factors or health measures between caregivers who reported follow-up data vs those who did not.

**Table 1.** Participant Characteristics at Baseline

Characteristic	No. (%)	
	Resource Sheet (n = 302)	Resource Sheet With Navigator Intervention (n = 309)
**Child**		
Age, mean (SD), y	6.1 (5.1)	6.1 (4.9)
Girls	152 (50.3)	163 (52.8)
Race/ethnicity		
Non-Hispanic white	11 (3.6)	9 (2.9)
Hispanic	240 (79.5)	243 (78.6)
Non-Hispanic black	22 (7.2)	32 (10.4)
Other or mixed	29 (9.7)	25 (8.1)
General health status rating, mean (SD)^a^	3.6 (1.0)	3.6 (1.0)
PedsQL functioning score, mean (SD)^b^		
Physical	82.8 (19.4)	82.2 (20.6)
Emotional	69.2 (23.4)	70.7 (21.1)
Social	82.2 (22.3)	80.6 (22.2)
Cognitive	84.3 (22.7)	89.4 (17.4)
School	73.5 (25.7)	69.1 (26.4)
Missed school days, mean (SD)	2.7 (3.1)	2.7 (3.3)
**Caregiver**		
Age, y		
18-24	38 (12.7)	36 (11.7)
25-34	109 (36.5)	125 (40.6)
35-44	117 (39.1)	117 (38.0)
45-74	35 (11.7)	30 (9.7)
Relationship to child		
Mother	264 (87.5)	276 (89.3)
Father	27 (8.9)	28 (9.1)
Other caregiver	11 (3.6)	5 (1.6)
Spanish-speaking	224 (74.2)	210 (68.0)
Race/ethnicity		
Non-Hispanic white	6 (2.0)	12 (3.9)
Hispanic	254 (84.1)	247 (79.7)
Non-Hispanic black	21 (7.0)	34 (11.0)
Other or mixed	21 (7.0)	16 (5.4)
Education level		
<High school	162 (53.7)	147 (47.8)
High school graduate or GED	71 (23.5)	101 (32.8)
Some college or college graduate	69 (22.8)	60 (19.4)
Household income <$35 000/y	242 (89.6)^c^	245 (87.8)^d^
Reported social risks, mean (SD), No.^e^	4.1 (3.2)	4.5 (3.1)
Self-rated general health status, mean (SD)^f^	2.8 (0.9)	2.8 (0.8)
Perceived Stress Scale score, mean (SD)^g^	5.6 (3.2)	5.8 (3.1)
Depression symptoms score, mean (SD)^h^	6.4 (5.3)	6.1 (4.8)

Abbreviations: GED, general educational development; PedsQL, Pediatric Quality of Life Inventory.

^a^ Range, 1 to 5, with higher score indicating better health.

^b^ Range, 0 to 100, with higher score indicating higher quality of life.

^c^ Includes data for 270 dyads.

^d^ Includes data for 279 dyads.

^e^ Range, 0 to 18.

^f^ Measured using the Patient-Reported Outcomes Measurement System short form version 1.1 (range, 1-5, with higher score indicating better health).

^g^ Range, 0 to 16, with higher score indicating more perceived stress.

^h^ Measured using the 8-item Patient Health Questionnaire (range, 0-24, with higher score indicating greater symptoms of depression).

The number of social risks reported at baseline ranged from 0 to 18 of 18 total possible items, and the mean (SD) was 4.3 (3.2) risks (Table 2). Of the 611 dyads, 47 dyads (7.7%) endorsed no social risks and 310 dyads (50.7%) reported 4 or more risks. At baseline, most caregivers reported child global health as excellent (123 dyads [21.6%]), very good (167 dyads [27.4%]), or good (246 dyads [40.3%]), 51 caregivers (8.4%) reported their child’s health was fair, and 14 caregivers (2.3%) reported their child’s health was poor. Caregivers characterized their own global health more negatively: 25 caregivers (4.1%) reported their global health was excellent, and 69 caregivers (11.3%) reported their global health was very good, while 307 caregivers (50.3%) reported good health, 179 caregivers (29.3%) reported fair health, and 30 caregivers (4.9%) reported poor health. The total number of social risk factors reported was inversely associated with child baseline health indicators and caregiver baseline health indicators (*r* = −0.23; *P* < .001), with a greater total number of social risk factors significantly associated with worse child health (*r* = −0.15; *P* < .001) and caregiver health (*r* = −0.23; *P* < .001), greater caregiver perceived stress (*r* = 0.31; *P* < .001), more caregiver depressive symptoms (*r* = 0.35; *P* < .001), more missed school days (*r* = 0.15; *P* < .001), and lower functioning scores on multiple dimensions of the PedsQL (physical: *r* = −0.18; *P* < .001; emotional: *r* = –0.26; *P* < .001; social: *r* = –0.19; *P* < .001; school: *r* = –0.24; *P* < .001). Frequencies of each social risk at baseline and 6 months after the intervention are presented in Table 2.

**Table 2.** Caregiver-Reported Social Risk Factors at Baseline and 6-Month Follow-up Stratified by Intervention Group

Social Risk	Resource Sheet Group (n = 225)				Resource Sheet With In-Person Assistance Group (n = 216)			
	No. (%)		Change, %	*P* Value	No. (%)		Change, %	*P* Value
	Baseline	6 mo			Baseline	6 mo		
Food insecurity	88 (39.1)	60 (26.6)	−12.4	<.001	77 (35.8)	51 (23.6)	−12.2	<.001
Unstable housing	94 (41.7)	47 (20.9)	−20.8	<.001	76 (35.2)	42 (19.4)	−15.7	<.001
Utility or telephone bills	83 (36.8)	51 (22.7)	−14.2	<.001	81 (37.5)	57 (26.4)	−11.1	.004
Housing quality	61 (27.1)	45 (20.0)	−7.1	.03	78 (36.1)	51 (23.8)	−12.3	.001
Difficulty finding job	63 (28.0)	34 (15.1)	−12.9	<.001	64 (29.6)	34 (15.7)	−13.9	<.001
Disability interfering with work	37 (16.5)	23 (10.2)	−6.3	.02	29 (13.4)	23 (10.6)	−2.8	.35
Problem with job	12 (5.4)	17 (7.6)	2.2	.41	23 (10.6)	13 (6.0)	−4.6	.10
Difficulty with unemployment insurance	19 (8.4)	8 (3.6)	−4.9	.03	16 (7.4)	10 (4.6)	−2.8	.24
Denied income support programs	29 (12.8)	30 (13.4)	0.6	.99	43 (19.9)	28 (12.9)	−7.0	.049
Health insurance	45 (20.0)	48 (21.3)	1.3	.77	53 (24.5)	45 (20.9)	−3.6	.35
Primary care clinician^a^	24 (12.4)	21 (10.9)	−1.6	.78	30 (17.2)	13 (7.5)	−9.7	.007
Medical or pharmacy bills	35 (15.6)	30 (13.3)	−2.2	.55	46 (21.3)	21 (9.8)	−11.5	.001
Afterschool activities	58 (26.0)	32 (14.3)	−11.7	<.001	70 (32.4)	20 (9.3)	−23.1	<.001
Childcare^a^	46 (23.8)	24 (12.5)	−11.3	<.001	48 (27.5)	21 (12.1)	−15.5	<.001
Bullying	24 (10.6)	19 (8.4)	−2.2	.42	34 (15.7)	21 (9.7)	−6.0	.05
Adult in household mental or behavioral health	43 (19.1)	23 (10.2)	−8.9	.003	42 (19.4)	30 (14.0)	−5.5	.15
Transportation	68 (30.2)	41 (18.3)	−11.9	<.001	64 (29.6)	37 (17.0)	−12.5	<.001
Other legal issues	60 (26.8)	46 (20.4)	−6.4	.76	71 (33.0)	52 (24.1)	−8.9	.02

^a^ This item was added after initial study initiation. Data for families missing this item at baseline were not included in 6-month follow-up analyses for this social risk factor or for analyses examining total number of social risks.

In the in-person assistance group, caregivers met with navigators a mean of 4.34 (SD, 2.76; range, 0-10) times after the initial visit, and 1228 of 1289 visits (95.3%) were conducted by telephone. Visit duration ranged from 5 to 45 minutes. Forty-five dyads (14.4%) did not engage in any navigation follow-up, and 35 dyads (11.2%) engaged in 8 or more contacts.

### Change in Social Risk Factors

There were no statistically significant differences between intervention groups in the number of social risk factors reported at follow-up with or without adjustment (Table 3). The number of successful navigator contacts was not significantly associated with decreases in number of social risks (*r* = −0.10; *P* = .15). In post hoc secondary analyses examining change within group, caregivers in the resource sheet group reported a significant decrease in number of social risks at 6-month follow-up relative to baseline with a mean (SE) difference of –1.28 (0.19) social risk factors (*P* < .001); caregivers in the in-person assistance intervention reported a mean (SE) change of –1.74 (0.21) social risk factors (*P* < .001) (Table 3). Among the individual social risk factors, significant improvement was seen on 13 of 18 risk factors within the in-person assistance intervention and 11 of 18 risk factors in the resource sheet intervention (Table 2). At follow-up, 192 caregivers in the resource sheet group (63.6%) and 191 caregivers in the in-person assistance group (61.8%) reported that their top social risk priority area was no longer an issue.

**Table 3.** Outcomes at 6-Month Follow-up Stratified by Intervention Group

Outcome	Resource Sheet, Mean (SE)			Resource Sheet With In-Person Assistance, Mean (SE)			Unadjusted Difference		Model 1 Adjusted Difference^a^		Model 2 Adjusted Difference^b^	
	Baseline	6 mo	Change	Baseline	6 mo	Change	Mean (SE) [95% CI]	*P* Value	Mean (SE) [95% CI]	*P* Value	Mean (SE) [95% CI]	*P* Value
Social risks endorsed^c^	3.95 (0.21)	2.66 (0.17)	−1.28 (0.19)	4.37 (0.20)	2.63 (0.20)	−1.74 (0.21)	−0.02 (0.16) [−0.28 to 0.33]	.86	−0.19 (0.22) [−0.61 to 0.24]	.39	−0.19 (0.22) [−0.62 to 0.23]	.92
**Child Health**												
Child general health	3.51 (0.07)	3.88 (0.06)	0.37 (0.07)	3.53 (0.07)	3.77 (0.06)	0.24 (0.07)	0.10 (0.09) [−0.07 to 0.27]	.25	0.11 (0.08) [−0.04 to 0.27]	.16	0.12 (0.09) [−0.04 to 0.27]	.13
Pediatric Quality of Life Inventory score^d^												
Physical functioning	82.97 (1.29)	85.64 (1.28)	2.67 (1.63)	81.87 (1.36)	84.18 (1.39)	2.31 (1.71)	1.26 (1.90) [−2.45 to 4.98]	.51	1.23 (1.85) [−2.41 to 4.87]	.51	1.58 (1.85) [−2.03 to 5.19]	.39
Emotional functioning	69.17 (1.57)	74.99 (1.35)	5.82 (1.37)	70.19 (1.44)	74.54 (1.23)	4.35 (1.26)	0.45 (1.83) [−3.15 to 4.03]	.81	.94 (1.52) [−2.04 to 3.93]	.53	1.40 (1.51) [−1.55 to 4.26]	.35
Social functioning	81.87 (1.50)	83.27 (1.34)	1.39 (1.37)	79.90 (1.54)	80.58 (1.46)	0.68 (1.74)	2.68 (1.98) [−1.19 to 6.57]	.18	1.90 (1.79) [−1.61 to 5.42]	.29	2.20 (1.80) [−1.32 to 5.73]	.22
Cognitive functioning^e^	84.30 (3.10)	88.76 (1.90)	4.46 (2.89)	90.14 (2.78)	85.74 (3.21)	−4.29 (4.18)	4.10 (3.58) [−2.91 to 11.12]	.25	4.06 (3.56) [−3.00 to 11.11]	.26	4.99 (3.56) [−1.99 to 11.96]	.16
School functioning^f^	73.86 (2.46)	72.43 (2.38)	−1.42 (2.41)	68.15 (2.31)	71.31 (2.31)	3.16 (2.52)	−0.41 (2.86) [−6.03 to 5.20]	.89	−1.43 (2.99) [−7.32 to 4.47]	.63	−0.49 (2.94) [−6.25 to 5.28]	.87
Missed school or camp d in past 6 mo, No.	2.82 (0.23)	2.12 (0.20)	−0.70 (0.25)	2.80 (0.24)	2.38 (0.22)	−0.42 (0.22)	−0.36 (0.28) [−0.91 to 0.19]	.20	−0.27 (0.31) [−0.79 to 0.27]	.31	−0.27 (0.27) [−0.79 to 0.25]	.31
**Caregiver Health**												
General health score^g^	2.87 (0.06)	3.00 (0.07)	0.14 (0.07)	2.80 (0.5)	2.97 (0.6)	0.17 (0.06)	0.03 (0.09) [−0.14 to 0.22]	.68	0 (0.08) [−0.16 to 0.16]	.98	−0.01 (0.08) [−0.17 to 0.15]	.90
Perceived Stress Scale score^h^	5.61 (0.21)	5.02 (0.22)	−0.59 (0.21)	5.73 (0.20)	4.75 (0.21)	−0.99 (0.22)	0.27 (0.30) [−0.87 to 0.31]	.36	0.34 (0.21) [−0.19 to 0.86]	.21	0.38 (0.27) [−0.90 to 0.15]	.16
Depression symptoms score^i^	6.52 (0.36)	4.82 (0.33)	−1.71 (0.31)	5.96 (0.31)	4.35 (0.29)	−1.60 (0.26)	0.45 (0.45) [−0.42 to 1.33]	.30	0.13 (0.35) [−0.56 to 0.81]	.72	0.16 (0.34) [−0.51 to 0.83]	.63

^a^ Calculated as the difference between groups in the outcomes at 6-month follow-up after adjusting for baseline outcomes.

^b^ Adjusted for model 1 and additional baseline covariates.

^c^ Range, 0 to 18.

^d^ Range, 0 to 100, with higher score indicating higher quality of life.

^e^ Includes data for 102 children based on applicability to specific age groups.

^f^ Includes data for 246 children based on applicability to specific age groups.

^g^ Measured using the Patient-Reported Outcomes Measurement System short form version 1.1 (range, 1-5, with higher score indicating better health).

^h^ Range, 0-16, with higher score indicating more perceived stress.

^i^ Measured using the 8-item Patient Health Questionnaire (range, 0-24, with higher score indicating greater depression symptoms).

### Change in Child Health

At follow-up, there were no statistically significant differences between intervention groups in the child health outcomes with or without adjustment (Table 3). Post hoc analyses indicated that caregiver report of child global health significantly improved (mean [SE]: written resources, 0.37 [0.07]; *P* < .001; in-person assistance, 0.24 [0.07]; *P* < .001). Similarly, both groups showed statistically significant improvement in caregiver report of children’s emotional functioning scores (mean [SE]: written resources, 5.82 [1.37]; *P* < .001; in-person assistance, 4.35 [1.26]; *P* < .001) and missed school or camp days (mean [SE]: written resources, −0.70 [0.25] days; *P* = .006; in-person assistance, −0.42 [0.22] days; *P* = .048) (Table 3). There were no significant changes in children’s physical, social, or cognitive or school functioning based on the PedsQL in either group.

### Change in Caregiver Health

Similar to the child health measures, there were no statistically significant differences at follow-up in caregiver general health, depression symptoms, or perceived stress between intervention groups with or without adjustment (Table 3). Post hoc analyses indicated that at follow-up, caregivers in both interventions reported significant improvement in their own general health (mean [SE]: written resource, 0.14 [0.07]; *P* = .04; in-person assistance, 0.17 [0.07]; *P* = .005), decreases in their perceived stress (mean [SE]: written resource, −0.59 [0.21]; *P* < .001; in-person assistance, −0.99 [0.22]; *P* < .001), and decreases in their reported depressive symptoms (mean [SE]: written resource, −1.71 [0.31]; *P* < .001; in-person assistance, −1.60 [0.26]; *P* < .001). Limiting the in-person assistance analytic sample to participants with 1 or more successful postenrollment contacts with a navigator resulted in a nearly identical pattern of results.

## Discussion

This medium-sized randomized clinical trial examined the effects of 2 clinical interventions designed to reduce families’ social and economic risks, a standardized handout that included information on available clinic, community, and government resources compared with the handout plus social services navigation assistance provided by a trained navigator for up to 3 months. There were no significant differences in the degree of change in social risk factors or child or caregiver health between the 2 interventions. Analyses within each trial arm showed significant decreases in reported social risk factors and improvements in both child and caregiver health from before to after the intervention. Social risk factors showing substantial reductions in both intervention groups included food insecurity, unstable or low-quality housing, problems paying bills, and difficulty affording transportation. These topics overlap with domains covered in common social risk screening tools endorsed by major professional organizations.^66,67^

There have been several other trials in pediatric settings that examine the effectiveness of multidomain social risk screening and interventions,^22,23,24^ although we are aware of only 1 that has included an intervention limited to providing written social resource information.^25^ In this randomized clinical trial, the lack of significantly greater benefit when adding longitudinal, in-person navigator services to the delivery of written social resources information was unexpected, given that an earlier trial in this area^25^ found significant differences between groups. Subtle but potentially important differences between these studies could help explain the disparate findings. It is possible that results of this study were affected by historical context,^68^ including increased federal immigration enforcement and anti-immigrant rhetoric. This might have lowered the likelihood of risk disclosure or assistance-seeking behaviors, although the interaction is unlikely to have differentially affected trial groups. A second difference between studies may be the quality of the information contained in the resource sheets. Handouts used in the prior trial^25^ relied on information developed by a county-contracted information and referral service and were updated only occasionally. In contrast, handouts used in this study were compiled by the navigators and included more regularly updated information about local social service providers. Handouts also incorporated 2 techniques recommended by the Agency for Healthcare Research and Quality to increase the usability of patient educational materials: adding contact names at relevant agencies and highlighting the resources most relevant to caregiver priorities.^69^ The provision of more up-to-date, targeted information may have increased the effect of the handouts in the written resources–only group of this trial relative to the effect in the prior trial.^25^ In brief, the greater the usefulness of the written resources, the less benefit may have been added by providing additional navigator services.

### Limitations

There are multiple limitations of this study. First, the study was conducted in only 1 urgent care setting, and it is unclear whether our findings are generalizable to other locations and clinical settings. Second, 27.8% of study participants were lost to follow-up at 6 months. Notably, this represents less attrition than in comparable studies undertaken in low-resource settings.^22,25,64,65^ Moreover, attrition did not differ between study groups and was not associated with key participant characteristics. Third, it is possible that the gift cards provided as part of the study influenced the positive postintervention outcomes in each group, although these should not have influenced the primary null findings between groups. Fourth, the surveys are based on participant self-report; they are therefore subject to classic respondent bias, which could have included underreporting or overreporting of social risks or health status. Fifth, the study compared the effect of different doses of a clinic-based intervention needed to decrease social risks. It would have been desirable to have a no-treatment control group to determine whether either or both levels of intervention significantly improved outcomes compared with screened but untreated controls. We did not add this condition owing to ethical concerns about assessing social risks without responding to endorsed needs.

## Conclusions

This randomized clinical trial of pediatric urgent care social risk interventions revealed no significant differences between provision of high-quality, personalized social service written information with vs without the addition of 1-on-1 longitudinal navigation services. The health care sector’s broad enthusiasm around activities to reduce patients’ social risks has contributed to an urgent need to identify effective and scalable interventions, particularly in pediatric settings where innovation in this area has outpaced the availability of evidence. However, counterbalancing this enthusiasm are the costs of screening and referral programs. The finding of this randomized clinical trial of pediatric urgent care social risk interventions should be encouraging to systems that are considering these kinds of programs. These findings suggest that a relatively low-dose and lower-cost intervention—high-quality, written information provided in the context of a single encounter—could be efficacious for pediatric populations, but similar interventions should be examined in studies designed to assess their effectiveness.

## Notes

Supplement 1. **Trial Protocol**

Supplement 2. **eAppendix.** Social Risk Screening

Supplement 3. **Data Sharing Statement**
