# Supplementary material for: Effects of In-Person Assistance vs Personalized Written Resources About Social Services on Household Social Risks and Child and Caregiver Health: A Randomized Clinical Trial
Source: JAMA Netw Open. 2020 Mar 10;3(3):e200701. doi: 10.1001/jamanetworkopen.2020.0701 (PMC7064877; doi:10.1001/jamanetworkopen.2020.0701)
Supplement: Supplement 1. — Trial Protocol [file jamanetwopen-3-e200701-s001.pdf]

Study Application (Version 1.29)

1.0 General Information

***Enter the full title of your study:**

SFGH Health Advocates Phase II Study

***Enter the study number or study alias**

SFGH Health Advocates Biomarkers
* This field allows you to enter an abbreviated version of the Study Title to quickly identify this study.

2.0 Add Department(s)

2.1 List the departments associated with this study. The Principal Investigator's department should be Primary.:

Primary Dept?	Department Name
◆ (selected)	**UCSF** - *113080* - M_Ctr for Health & Community
○	**UCSF** - *126612* - M_FCM-FamMed-ZSFG-Programs

3.0 List the key study personnel: (Note: external and affiliated collaborators who are not in the UCSF directory can be identified later in the Qualifications of Key Study Personnel section at the end of the form)

3.1 *Please add a Principal Investigator for the study:

Gottlieb, Laura, MD, MPH

Select if applicable

☐ Department Chair ☐ Resident

☐ Fellow

If the Principal Investigator is a Fellow, the name of the Faculty Advisor must be supplied below.

3.2 If applicable, please select the Research Staff personnel:

A) Additional Investigators

Adler, Nancy E PhD, PhD
Other Investigator
Boyce, William MD
Other Investigator
Hessler, Danielle, PhD
Other Investigator
Keeton, Victoria

Other Investigator
Pantell, Matthew S
Other Investigator

B) Research Support Staff

Arevalo, Catherine
Research Assistant
Aronstam, Alison L
Research Assistant
Chernitskiy, Stephanie L
Study Coordinator
De Vore, Joanna D
Study Coordinator
Hernandez, Maricarmen
Research Assistant
Herrera, Philip H
Research Assistant
Munoz, Andrea K
Research Assistant
Romero, Abigail
Research Assistant
Urrutia Caceres, Elizabeth
Research Assistant
Velazquez, Denisse E
Research Assistant
Wing, Holly E
Study Coordinator
Yee, Megan D
Volunteer/Student Intern (through UCSF Medical Center)

3.3 *Please add a Study Contact:

Chernitskiy, Stephanie L
Gottlieb, Laura, MD, MPH
Wing, Holly E

The Study Contact(s) will receive all important system notifications along with the Principal Investigator. (e.g. The project contact(s) are typically either the Study Coordinator or the Principal Investigator themselves).

3.4 If applicable, please add a Faculty Advisor/Mentor:

3.5 If applicable, please select the Designated Department Approval(s):

Add the name of the individual authorized to approve and sign off on this protocol from your Department (e.g. the Department Chair or Dean).

4.0 Qualifications of Key Study Personnel

4.1 November, 2015 - NEW Definition of Key Study Personnel and CITI Training

Requirements:

UCSF Key Study Personnel include the Principal Investigator, other investigators and research personnel who are directly involved in conducting research with study participants or who are directly involved in using study participants' identifiable private information during the course of the research. Key Personnel also include faculty mentors/advisors who provide direct oversight to Postdoctoral Fellows, Residents and Clinical Fellows serving as PI on the IRB application. **The IRB requires that all Key Study Personnel complete Human Subjects Protection Training through CITI prior to approval of a new study, or a modification in which KSP are being added. More information on the CITI training requirement can be found on our website.**

List the study responsibilities and qualifications of any individuals who qualify as Key Study Personnel (KSP) at UCSF and affiliated sites ONLY by clicking the "Add a new row" button. This information is required and your application will be considered incomplete without it.

KSP Name	Description of Study Responsibilities	Qualifications
Gottlieb, Laura, MD, MPH	Initiate study concept, contribute to study design, data analyses and writing up study results.	Dr. Gottlieb is a clinician and social determinants of health expert with experience designing and implementing studies in this arena.
Dr. Adler, Nancy E PhD, PhD	Will contribute to all aspects of study design and writing up study results.	Dr. Adler has over 30 years experience in scientific study design around social determinants of health.
Hessler, Danielle, PhD	Responsible for data analysis and writing up study results.	Dr. Hessler has been the statistician and key study team member on multiple large scientific analyses. She is co-PI on a similar study at CHRCO and holds a PhD in developmental psychology.
Dr. Boyce, William MD	Supervise biomarker analyses.	Dr. Boyce is a nationally renowned physician researcher who has pioneered biomarkers as a key physiological window into stress' impacts on health.
Wing, Holly E	Responsible for creating and maintaining study protocols, coordinating with SFGH urgent care clinic and health advocate program.	Ms. Wing has several years experience coordinating research studies at UCSF and SFGH.
Herrera, Philip H	Responsible for assisting RA with recruitment, data and biomarker collection and randomizing participants into research groups and implementing intervention.	Mr. Herrera has trained as an SFGH health advocate and has worked as a research assistant and community health worker. Mr. Hererra is bilingual (Spanish and English).
De Vore, Joanna D	Responsible for recruiting participants, collecting survey data and biomarkers and following up with participants.	Ms. De Vore has 6 + years working as a Clinical Research Coordinator at UCSF. She has experience recruiting patients in clinical setting and administering surveys. She is bilingual (Spanish and English).

Velazquez, Denisse E	Responsible for assisting RA with recruitment, data and biomarker collection and randomizing participants into research groups and implementing intervention.	Ms. Velazquez has 2+ years working as a health advocate at SFGH. She has worked as a research assistant and is bilingual (Spanish and English).	
Yee, Megan D	The research volunteer will observe recruitment and follow-up study activities in clinic to enrich her learning experience. Study and data management learning opportunities will also be made available.	Ms. Yee has 2+ years volunteering in health clinics and on research studies.	
Romero, Abigail	Responsible for assisting RA with recruitment, data and biomarker collection and randomizing participants into research groups and implementing intervention.	Ms. Romero has served has 2+ years working as a health advocate at SFGH. During that time, she surveyed participants as part of the first Health Advocate study. She is bilingual (Spanish and English).	
Urrutia Caceres, Elizabeth	Responsible for assisting RA with recruitment, data and biomarker collection and randomizing participants into research groups and implementing intervention.	Ms. Urrutia is a SF Build scholar working with our team to receive research training. She has worked as a health educator within our patient population and is bilingual (Spanish & English).	
Arevalo, Catherine	Responsible for assisting RA with recruitment, data and biomarker collection and randomizing participants into research groups and implementing intervention.	Ms. Arevalo has served for 6 months as a health advocate at ZSFG. During that time, she surveyed and worked with our patient population. She is bilingual (Spanish and English).	
Munoz, Andrea K	Responsible for assisting RA with recruitment, data and biomarker collection and randomizing participants into research groups and implementing intervention.	Ms. Munoz has served for 6 months as a health advocate at ZSFG. During that time, she surveyed and worked with our patient population. She is bilingual (Spanish and English).	
Hernandez, Maricarmen	Responsible for assisting RA with recruitment, follow-up interviews and data and biomarker collection.	Ms. Hernandez has 1+ years working as a research assistant and 1+ years experience working in safety net clinics serving our patient populations. She is bilingual (Spanish and English).	
Keeton, Victoria	Responsible for study operations within ZSFG pediatric urgent care clinic. Will add to study design.	Ms. Keeton is a certified pediatric nurse practitioner and clinical nurse specialist with 12 years of experience in pediatric primary care and 11 years as a nurse educator. She has primarily served Latino and under-resourced populations in community health.	
Aronstam, Alison L	Develop qualitative interview.	Ms. Aronstam is a student in the UC Berkeley-UCSF joint	

		medical program.
Pantell, Matthew S	Dr. Pantell will assist with data analysis and writing up findings for publication.	Dr. Pantell has extensive statistical analysis experience.

5.0 Initial Screening Questions - Updated 9/13

(Note: You must answer every question on this page to proceed). If you are converting to the new form, check questions 5.4, 5.6, 5.7, 5.8 and 5.10 before saving and continuing to the next section.

5.1 * Application type:

- ◯ Full Committee
- ◉ Expedited
- ◯ Exempt

5.2 * Risk level (Help Text updated 9/13):

- ◉ Minimal risk
- ◯ Greater than minimal risk

5.3 * Subject contact:

- ◉ Yes (including phone, email or web contact)
- ◯ No (limited to medical records review, biological specimen analysis, and/or data analysis)

5.4 * Funding (past or present):

- ◉ Funded or will be funded (external sponsor, gift, program or specific internal or departmental funds)
- ◯ Unfunded (no specific funds earmarked for this project)
- ◯ Unfunded student project

5.5 * The Principal Investigator and/or one or more of the key study personnel has financial interests related to this study:

◯ Yes ◉ No

If **Yes**, the Conflict of Interest Advisory Committee (COIAC) office may contact you for additional information.

5.6 * This is an investigator-initiated study:

◉ Yes ◯ No

5.7 * This study ONLY involves retrospective records review and/or identifiable biospecimen analysis:

◯ Yes ◉ No

5.8 * This is a clinical trial:

(•) Yes ( ) No

Clinical Trial Registration
"NCT" number for this trial:

NCT02746393

5.9 * This is a multicenter study:

( ) Yes (•) No

5.10 * This application involves the study of unapproved or approved drugs, devices, biologics or in vitro diagnostics:

( ) Yes (•) No

5.11 * This application involves a Humanitarian Use Device:

(•) No
( ) Yes, and it includes a research component
( ) Yes, and it involves clinical care ONLY

5.12 * This study involves human stem cells (including iPS cells and adult stem cells), gametes or embryos:

(•) No
( ) Yes, and requires CHR and GESCR review
( ) Yes, and requires GESCR review, but NOT CHR review

5.13 * This is a CIRB study (e.g. the NCI CIRB will be the IRB of record):

( ) Yes (•) No

5.14 * This application includes a request to rely on another IRB (other than NCI CIRB):

( ) Yes (•) No

Note: If this request is approved, the CHR will **NOT** review and approve this study. Another institution will be the IRB of record.

6.0 Expedited Review Categories

6.1 * If you think this study qualifies for expedited review, select the regulatory category(ies) that the research falls under:

- [ ] Category 1: A very limited number of studies of approved drugs and devices
- [ ] Category 2: Blood sampling
- [x] Category 3: Noninvasive specimen collection (e.g. buccal swabs, urine, hair and nail clippings, etc.)
- [ ] Category 4: Noninvasive clinical procedures (e.g. physical sensors such as pulse oximeters, MRI, EKG, EEG, ultrasound, moderate exercise testing, etc.)
- [ ] Category 5: Research involving materials (data, documents, records, or specimens) that were previously collected for either nonresearch or research purposes
- [ ] Category 6: Use of recordings (voice, video, digital or image)

☑ Category 7: Low risk behavioral research or research employing survey, interview, oral history, focus group, program evaluation, human factors evaluation, or quality assurance methodologies

7.0 Funding

7.1 Identify all sponsors and provide the funding details. If funding comes from a Subcontract, please list only the Prime Sponsor: **Note: we require only a P Number *OR* an A Number for funding coming through UCSF. Please avoid these common errors in funding documentation:**

- **DO NOT add the A Number if a P Number was already provided OR update the A Number field when a new funding cycle begins. The IRB does NOT use this information or want these changes made.**
- **DO NOT add a grant continuation as a new funding source.**

External Sponsor:

View Details	Sponsor Name	Sponsor Type	Awardee Institution	Contract Type:	UCSF RAS "P number" or eProposal number	UCSF RAS System Award Number ("A" + 6 digits)
⊟	JPB Foundation, The	07	UCSF	Subcontract	P0527438	

Sponsor Name:	JPB Foundation, The
Sponsor Type:	07
Sponsor Role:	Funding
**Grant/Contract Number:**	
Awardee Institution:	UCSF
**Is Institution the Primary Grant Holder:**	No
**if No, then who is the Primary Grantee?**	
Contract Type:	Subcontract
UCSF RAS "P number" or eProposal number:	P0527438
UCSF RAS System Award Number ("A" + 6 digits):	
Grant Number for Studies Not Funded thru UCSF:	
Grant Title:	Research Network on Toxic Stress and Health
PI Name:<br>(If PI is not the same as identified on the study.)	William Thomas Boyce, MD
Significant Discrepancy:	

View Details	Sponsor Name	Sponsor Type	Awardee Institution	Contract Type:	UCSF RAS "P number" or eProposal number	UCSF RAS System Award Number ("A" + 6 digits)
⊟	JPB Foundation, The	07	UCSF	Subcontract	P0537151	

Sponsor Name:	JPB Foundation, The
Sponsor Type:	07
Sponsor Role:	Funding

**Grant/Contract Number:**	
Awardee Institution:	UCSF
**Is Institution the Primary Grant Holder:**	Yes
Contract Type:	Subcontract
UCSF RAS "P number" or eProposal number:	P0537151
UCSF RAS System Award Number ("A" + 6 digits):	
Grant Number for Studies Not Funded thru UCSF:	
Grant Title:	Research Network on Toxic Stress and Health
PI Name:<br>(If PI is not the same as identified on the study.)	Nicki Bush
Significant Discrepancy:	

Gift, Program, or Internal Funding (check all that apply):

☑ Funded by gift (specify source below)
☑ Funded by UCSF or UC-wide program (specify source below)
☐ Specific departmental funding (specify source below, if applicable)

List the gift, program, or departmental funding source:

Pritzker Family Fund; Harvard Research Network; RAPtr; UCB Joint Medical Program

7.2 If you tried to add a sponsor in the question above and it was not in the list, follow these steps:

- **If funding has already been awarded or the contract is being processed by the Office of Sponsored Research (OSR) or Industry Contracts Division (ICD), your sponsor is already in the system and the project has an eProposal Proposal or Award number. Check with your department's OSR Staff or ICD Officer to ask how the sponsor is listed in the UC sponsor list and what the Proposal or Award number is. Click here to find your OSR staff and here to find your ICD staff.**
- **If your sponsor is not yet in the list, enter it in the box below.**

◉ Sponsor not in list

Only if your sponsor is not yet in the list, type the sponsor's name:

Lisa and John Pritzker Family Fund; Harvard Research Network on Toxic Stress

If the funding is administered by the UCSF Office of Sponsored Research, your study will not receive CHR approval until the sponsor and funding details have been added to your application.

7.3 * This study is currently supported in whole or in part by Federal funding OR has received ANY Federal funding in the past (Help Text updated 9/13):

○ Yes ◉ No

If **yes**, indicate which portion of your grant you will be attaching:

☐ The Research Plan, including the Human Subjects Section of your NIH grant or subcontract
☐ For other federal proposals (contracts or grants), the section of the proposal describing human

subjects work

- [ ] The section of your progress report if it provides the most current information about your human subjects work
- [ ] The grant is not attached. The study is funded by an award that does not describe specific plans for human subjects, such as career development awards (K awards), cooperative agreements, program projects, and training grants (T32 awards) OR UCSF (or the affiliate institution) is not the prime recipient of the award

8.0 Sites

8.1 Institutions (check all that apply):

- [ ] UCSF
- [ ] China Basin
- [ ] Helen Diller Family Comprehensive Cancer Center
- [ ] Mission Bay
- [ ] Mount Zion
- [x] San Francisco General Hospital (SFGH)
- [ ] SF VA Medical Center (SF VAMC)
- [ ] Blood Centers of the Pacific (BCP)
- [ ] Blood Systems Research Institute (BSRI)
- [ ] Fresno (Community Medical Center)
- [ ] Gallo
- [ ] Gladstone
- [ ] Institute on Aging (IOA)
- [ ] Jewish Home
- [ ] SF Dept of Public Health (DPH)

8.2 Check all the other types of sites not affiliated with UCSF with which you are cooperating or collaborating on this project (Help Text updated 9/13):

- [x] Other UC Campus
- [x] Other institution
- [ ] Other community-based site
- [ ] Foreign Country

List the foreign country/ies:

8.3 Check any research programs this study is associated with:

- [ ] Cancer Center
- [ ] Center for AIDS Prevention Sciences (CAPS)
- [ ] Global Health Sciences
- [ ] Immune Tolerance Network (ITN)
- [ ] Neurosciences Clinical Research Unit (NCRU)
- [ ] Osher Center
- [ ] Positive Health Program

9.0 Studies Involving Other Sites

9.1 UCSF is the coordinating center:

◉ Yes ○ No

If **Yes**, describe the plan for communicating safety updates, interim results, and other information that may impact risks to the subject or others among sites:

All study activities will take place at ZSFG hospital. No participants will be consented, enrolled or participate in study activites at UC Berkeley. Alison Aronstam, a member of our study team is based at UC Berkeley. Ms. Aronstam will receive supervision and mentorship from her UC Berkeley advisors throughout the study, but those advisors will not have any contact with research subjects through recruitment, consenting, interviewing, or reviewing indentifiable data.

Analysis and Interpretation of Biological Data. Biological specimens collected as part of this research will be analyzed and stored at Dr. Michael Meaney's lab at the Research Center of the Douglas Mental Health University Institute, McGill University in Montreal, Canada. De-identified patient-level survey data collected during the course of the study will be transferred securely to Dr. Michael Meaney's lab at the Research Center of the Douglas Mental Health University Institute, McGill University for use in the interpretation of the biological data analyses. Transfer of patient-level survey data and biological samples are clearly stated in a data sharing agreement with the Research Center of the Douglas Mental Health University Institute, McGill University.

If **Yes**, describe the plan for sharing modification(s) to the protocol or consent document(s) among sites:

See above.

9.2 Check any other UC campuses with which you are collaborating on this research study:

- [x] UC Berkeley
- [ ] UC Davis
- [ ] Lawrence Berkeley National Laboratory (LBNL)
- [ ] UC Irvine
- [ ] UC Los Angeles
- [ ] UC Merced
- [ ] UC Riverside
- [ ] UC San Diego
- [ ] UC Santa Barbara
- [ ] UC Santa Cruz

9.3 Are the above UC campuses requesting to rely on UCSF's IRB (check all that apply):

- [x] Yes (Submit a reliance request through the UC IRB Reliance Registry)
- [ ] No (Complete IRB Approval Certification section)

10.0 Outside Site Information

10.1 Outside Site Information

Click "Add a new row" to enter information for a site. Click it again to add a second site again to add a third site, a fourth site, etc.

Outside Site Information

Non-UCSF affiliated site information:

Site name:

Research Center of the Douglas Mental Health University Institute, McGill Univer

Contact name:

Michael Meaney

Email:

michael.meaney@mcgill.ca

Phone:

(514) 761-6131 x3938

For Federally-funded studies only, corresponding FWA#:

*** The research at this site will be reviewed by:**

- (selected) The non-affiliated site's IRB or a private IRB
- The non-affiliated site is requesting UCSF to be the IRB of record for this study
- The non-affiliated site is not engaged in the human subjects research and has provided a letter of support

If the other site's IRB approval letter is available now, attach it to the application. If the IRB approval letter is not yet available, submit it once you receive it.

Or, if the other site is **not engaged** in human subjects research, attach the letter of support to your application.

11.0 Study Design

11.1 * Study design (Help Text updated 9/13):

We are proposing an 18-month RCT that collects survey and non-invasive biological data from caregivers accompanying children who are visiting a pediatric urgent care center. Our previous, similar RCT study data collection ended on 8/31/15 as scheduled. Though the social needs-focused intervention is now a routine, standardized part of the pediatric primary care setting, we are proposing a new urgent care-focused randomized controlled trial where we will enroll eligible patients solely from urgent care. In one arm, we will offer the intervention; in the other, we will offer written information on available community resources, which is already above the standard of care. All patients in both arms will be followed over a six month period to capture information about changes in their social needs and health outcomes. Participants will be randomized into intervention or control arms of the study using a stratified randomization method in the computer-based program REDCap.

Activities: Following consent at time of enrollment, at both baseline and 6 mo follow up, we will administer a survey and collect hair, saliva and buccal mucosal cell swabs (from child and adult caregiver).

11.2 If this is a clinical trial, check the applicable phase(s) (Help Text updated 9/13):

- [x] Phase I
- [ ] Phase II
- [ ] Phase III
- [ ] Phase IV

12.0 Scientific Considerations

12.1 Hypothesis (Help Text updated 9/13):

This study has a hypothesis:

(•) Yes ( ) No

If yes, state the hypothesis or hypotheses:

We hypothesize that participants in the SFGH Health Advocacy program will experience improved physical and emotional health (child); improved caregiver mental health; decreases in hair cortisol (child and caregiver); fewer missed school (child) or work days (caregiver): and decreases in acute care utilization (child). Additional outcomes of interest include other stress biomarkers, like salivary inflammatory cytokines and DNA genotyping and methylation.

12.2 * List the specific aims:

This research phase evaluates the health impacts of the SFGH Heatlh Advocates program. In addition to the aims of the first RCT we conducted from 2013-2015, we will measure the effects of the program on:

1. Child health measures
2. Child and caregiver stress and immunologic biomarkers, including hair cortisol levels.
3. Caregiver stress, depression and social support.
4. Caregiver health status.

12.3 Statistical analysis:

Descriptive Analyses & Missing Data. Descriptive analyses will include family demographics, biomarker, child and caregiver health, and family social needs. Data will be inspected for outliers and out-of-range values. Examination of distributions may prompt transformations, where they are defensible and are a component of the best available analysis strategy. Although protocols have been designed to promote retention, some attrition is inevitable. We will perform analyses to determine whether differential attrition occurred by patient characteristics. Likelihood based approaches (e.g. GLMMs) will be used to handle missing data. This approach fits models to all available data and invoke the relative assumption that the data are missing at random.

Background demographics will be examined to describe the sample and will be included in multivariate analysis if they are related to the outcome at p< .2 or associated with dropout. Correlations among variables will be examined and redundant measures will be combined or eliminated to avoid multicollinearity. We will compare participants randomized to the two groups on caregiver and child characteristics using t-tests and chi-square tests.

Assessment of Outcomes: We will fit regression models for cross-sectional and longitudinal outcomes. Changes from baseline to follow-up on the primary outcomes will be estimated with linear mixed models and generalized linear models with generalized estimating equations (e.g., SAS PROC MIXED ROC GENMOD, SAS Institute Inc., 1999). Generally, we seek to model longitudinally the trajectory of the primary outcomes as a function of time. We begin by fitting *base* models, those with intervention group and minimum covariates and, for longitudinal models, the most restricted residual covariance structures. We will then consider additional baseline demographic or family predictors, and baseline family predictors-by-time interaction in subsequent models to examine moderators of change. Similar models will be used to examine each of the primary outcomes outlined. Empirical contributions to model selection decisions will include reference to information criteria (e.g., Akaike's). We will examine change from baseline for each outcome as the dependent variable.

12.4 If this study has undergone scientific or scholarly review, please indicate which entity performed the review:

☐ Cancer Center Protocol Review Committee (PRC) (Full approval is required prior to final CHR approval for cancer-related protocols.)
☐ CTSI Clinical Research Center (CRC) advisory committee
☐ Departmental scientific review
☐ Other:

Specify **Other**:

13.0 Background

13.1 Background:

Adverse social circumstances like hunger and food insecurity can have dramatic, negative impacts on the health of vulnerable children.(1-5) In safety-net settings, the prevalence of these adverse social circumstances are alarmingly high.(6-7) A 2007 study from Boston Medical Center pediatric emergency department found that over 97% of presenting families had at least one basic social need; over 48% of families reported that within the last 12 months they had either been threatened with or experience utility shut-offs; and over 30% of families said that they had either reduced the size of their meals or skipped meals because they did not have enough money for food.(8) In a study in pediatric urban primary care clinics, over 82% of families reported a least one social need.(9)

Despite the preponderance of social need in both emergency room and primary care safety net settings, the current standard of practice in pediatric medicine fails to address many of the health-related social problems of struggling families. Garg et al. (2007) showed significant gaps between primary care providers' beliefs that they should include social screening topics in pediatric visits and routinized social screening behaviors.(10) Other researchers have demonstrated that comprehensive social screening (across multiple social domains) is particularly rare in pediatric settings.(9)The evidence suggesting irregular and informal screening for health-related social needs belies studies demonstrating that conducting social screening informally during clinical encounters poorly estimates prevalence; providers routinely underestimate social needs in all areas except drug and alcohol use and need for nursing home placement.(11) Furthermore, standardized screening has been shown to be more sensitive than provider clinical interviews.(12)

There is growing evidence that patients who receive public benefits may have better health status and decreased emergency room utilization.(13-19). Despite this emerging literature that serves as important background for FIND implementation, existing intervention programs to address social factors in pediatric clinical settings (e.g. healthleadsusa.org) have not yet included scientific evaluations in either emergency room or primary care settings of program impacts on pediatric health care status or health care utilization, nor on patient satisfaction/connectedness to health care organizations. As a result of conducting a randomized controlled clinical trial, we hope to prove that by systematically addressing our families social determinants of health, we will better connect families to community resources, use healthcare resources more appropriately, improve health status and enhance patient satisfaction.

The San Francisco General Hospital (SFGH) Health Advocacy (HA) intervention specifically includes implementing a navigation desk at the SFGH pediatric urgent care center. The program is available to all families presenting for care to the SFGH Primary Care Clinic. All families entering the site are offered screening for social issues affecting their health, including nutrition, housing and financial insecurity. Families with a positive screen are referred to the HA program, where navigators assist them to address each family's specific needs. Using a client management database, HA navigators follow the family over time to ensure that identified needs have been successfully addressed. The initial RCT data collection effort of the HA intervention was very successful. Results showed that participants in the intervention arm decreased needs and improved child health significantly more than the control group arm (publication pending). In this new study phase, we plan to examine the effects of the HA program on a more robust set of survey data including detailed measures of caregiver and child health and non-invasive biological data. We will collect survey responses to a broader range of health outcomes for children and their caregivers, including intermediate health outcomes like caregiver depression, work and school attendance, and biomarkers of stress IL-6/ inflammatory cytokines (oral swab collection) and hair cortisol. This work is based on existing studies showing that hair cortisol and inflammatory cytokines are important markers of socioeconomic status and social stressors.(20-23)

13.2 Preliminary studies:

Patient surveys at SFGH have shown that almost 50% of families accessing care in the primary care clinics have experienced food insecurity over the last 12 months. This population is expected to be assisted with the SFGH HA intervention. The phase one RCT research on CCLiP showed that 50% of intervention families decreased by at least one social need and many decreased more than that; the study also shows a .35 point (on a five point scale) improvement on parent reported child health in comparison with the control group. These findings are comparable to other new studies showing that social needs interventions are successful at promoting referrals to community resources. (24)

13.3 References:

1. Larson K, Russ SA, Crall JJ, Halfon N. Influence of multiple social risks on children's health. Pediatrics. 2008;
121;337.
2. Wood PR, Smith LA, Romero D, Bradshaw P, Wise PH, Chavkin W. Relationships between welfare status, health insurance status and health and medical care among children with asthma. Am J Pub Health. Sep
2002;92(9):1146-1452.
3. Cook JT, Frank DA, Berkowitz C, Black MM, Casey PH, Cutts DB, Meyers AF, Zaldivar N, Shalicky A, Levenson S, Heeren T. Welfare reform and the health of young children: A sentinel survey in 6 US cities. Arch PediatrAdolesc Med Jul 2002;156:678-684.
4. Frank DA, Neault NB, Skalicky A, Cook, JT, Wilson JD, Levenson S, Meyers AF, Heeren T, Cutts DB, Casey PH, Black MM, Berkowitz C. Heat or eat: the Low Income Home Energy Assistance Program and nutritional health risks among childen less than 3 years of age. Pediatrics 2006 Nov;118(5):e1293-302.
5. Meyers A, Cutts D, Frank DA, et al. Subsidized housing and children's nutritional status: data from a multisite surveillance study. Arch Pediatr Adolesc Med. 2005 Jun;159(6):551-6.
6. Garg A, Butz AM, Dworkin PH, Lewis RA, Serwint JR. Screening for basic social needs at a medical home for low-income children. ClinPediatr.2009 48:32-36;
7. Lawton E, Leiter K, Todd J, Smith L. Welfare reform: advocacy and intervention in the health care setting. Pub
Health Reports. Nov/Dec 1999;114:540-549.
8. Hanson M, Lawton E. Between a rock and a hard place: The prevalence and severity of unmet legal needs in the pediatric emergency department setting. Medical Legal Partnership for Children. 2007.
9. Fleegler EW, Lieu TA, Wise PH, Muret-Wagstaff S. Families' health-related social problems and missed referral opportunities. Pediatrics. 2007;119:e1332–41.
10. Garg A, Butz AM, Dworkin PH, Lewis RA, Thompson RE, Serwint JR, Improving the management of family psychosocial problems at low-income children's well-child care visits: the WE CARE project. Pediatrics.
2007;120:547-558.
11. Bikson, K., McGuire, J.,Blue-Howells, J.&Seldin-Sommer, L. Psychosocial Problems in primary care: Patient and provider perceptions. Social Work in Health Care. 2009;48:736-749.
12. Keller, D., Jones, N., Savageau, J. A. &Cashman, S. B. Development of a brief questionnaire to identify families in need of legal advocacy to improve child health. Ambulatory Pediatrics. 2008;8:266-269.
13. Cook JT, Frank DA, Berkowitz C, Black MM, Casey PH, Cutts DB, Meyers AF, Zaldivar N, Skalicky A, Levenson S, Heeren T. Welfare reform and the health of young children: a sentinel survey in 6 US cities. Arch Pediatr Adolesc Med2002 Jul;156(7):678-84.
14. Frank DA, Neault NB, Skalicky A, Cook JT, Wilson JD, Levenson S, Meyers AF, Heeren T, Cutts DB, Casey PH, Black MM, Berkowitz C.Heat or eat: the Low Income Home Energy Assistance Program and nutritional and health risks among children less than 3 years of age. Pediatrics 2006 Nov;118(5):e1293-302.
15. Meyers A, Cutts D, Frank DA, Levenson S, Skalicky A, Heeren T, Cook J, Berkowitz C, Black M, Casey P, Zaldivar N. Subsidized housing and children's nutritional status: data from a multisite surveillance study. Arch Pediatr Adolesc Med 2005 Jun;159(6):551-6.
16. Black MM, Cutts DB, Frank DA, Geppert J, Skalicky A, Levenson S, Casey PH, Berkowitz C, Zaldivar N, Cook JT, Meyers AF, Herren T. Special Supplemental Nutrition Program for Women, Infants, and Children participation and infants' growth and health: a multisite surveillance study. Pediatrics 2004 Jul; 114(1):169-76.
17. Jones SJ, Jahns L, Laraia BA, Haughton B. Lower risk of overweight in school-aged food insecure girls who participate in food assistance: results from the panel study of income dynamics child development supplement. Arch Pediatr Adolesc Med 2003 Aug;157(8):780-4.
18. Cook JT, Frank DA, Berkowitz C, Black MM, Casey PH, Cutts DB, Meyers AF, Zaldivar N, Skalicky A, Levenson S, Heeren T, Nord M. Food insecurity is associated with adverse health outcomes among human infants and toddlers. J Nutr 2004 Jun;134(6):1432-8.
19. Cook JT, Frank DA, Levenson SM, Neault NB, Heeren TC, Black MM, Berkowitz C, Casey PH, Meyers AF, Cutts DB, Chilton M. Child food insecurity increases risks posed by household food insecurity to young children's health. J Nutr 2006 Apr;136(4):1073-6.

20. Staufenbiel SM, Penninx BW, Spijker AT, Elzinga BM, & van Rossum EF (2013). Hair cortisol, stress exposure, and mental health in humans: a systematic review. Psychoneuroendocrinology, 38(8):1220-. 1235.
21. Sharpley CF, McFarlane JR, & Slominski A (2012). Stress-. linked cortisol concentrations in hair: what we know and what we need to know. Reviews in the neurosciences, 23(1):111-. 121.
22. Steptoe A, Hamer M, & Chida Y (2007). The effects of acute psychological stress on circulating inflammatory factors in humans: a review and meta-analysis. Brain, behavior, and immunity 21(7): 901 912.
23. Weik U, Herforth A, Kolb-. Bachofen V, & Deinzer R (2008) Acute stress induces proinflammatory signaling at chronic inflammation sites. Psychosomatic medicine. 70(8):906-912.
24. Garg A, et al. Addressing Social Determinants of Health at Well Child Care Visits: A Cluster RCT. Pediatrics 2015;135;e296.

If you have a separate bibliography, attach it to the submission with your other study documents.

14.0 Sample Size and Eligibility

14.1 Number of subjects that will be enrolled at UCSF and affiliated institutions:

1448

14.2 Total number of subjects that will be enrolled at all sites (Help Text updated 9/13):

1448

14.3 Estimated number of people that you will need to consent and screen here (but not necessarily enroll) to get the needed subjects:

3000

14.4 Explain how and why the number of subjects was chosen (Help Text updated 9/13):

Total enrollment for this phase will not exceed 724 dyads (724 caregivers + 724 children) Power Analysis. Sample size and power estimates are based on alpha = 0.05 and 2-sided t-tests on between group differences in change from baseline to follow-up between the two groups. Estimates of means (SDs) at follow-up from related studies and our own previous work suggest effect sizes that are small to moderate, with 0.26 to .43 SD unit differences for primary parent-reported outcome of number of social needs and 0.38 to 0.46 SD unit differences for change in cortisol. A total sample of 724 dyads (n=362 per arm) will provide power to exceed 0.80 for tests on all primary outcomes. We conservatively estimate a 30% attrition rate over the course of the study, resulting in a final sample of 506 dyads post attrition (n=253 per arm). The study is powered at least 80% to detect small/medium effect sizes of d=. 25 or greater. This sample size allows at least 80% power to detect estimated mean differences of changes from baseline of magnitude 0.50 or greater for our likert based primary outcome of number of social needs.

14.5 * Eligible age range(s):

☑ 0-6 years
☑ 7-12 years
☑ 13-17 years
☑ 18+ years

14.6 Inclusion criteria:

- English or Spanish speaking parent or legeal gaurdian

- Parent or legal guardian accompanying an SFGH Primary Care or Urgent Care
- Clinic patient 0-17 years old
- Parent or legal guardian over or equal to 18 years old
- Parent or legal guardian resides with child in SF County
- Are not currently receiving services from the Health Advocate program or have not received Health Advocate services in the six months prior to enrollment

14.7 Exclusion criteria:

- Non-English or non-Spanish speaking parent or legal gaurdian
- Parent or legal guardian under age 18
- Parent or legal guardian accompanying patient is not familiar with the child's living situation
- Currently receiving services from the Health Advocate program or have received Health Advocate services in the past six months
- Non-San Francisco resident
- Foster child or child in clinic for a child protective clearance exam
- In clinic for exam related to child abuse
- Clinic staff

14.8 There are inclusion or exclusion criteria based on gender, race or ethnicity:

○ Yes ● No

If **yes**, please explain the nature and rationale for the restrictions:

15.0 Other Approvals and Registrations

15.1 * Do any study activities take place on patient care units:

● Yes ○ No

If **Yes**, attach a letter of support for the study from the involved patient care manager(s).

15.2 * Does your protocol involve any radiation exposure to patients/subjects? The UCSF Radiation Safety Committee requires review of your protocol if it includes administration of radiation as part of standard of care OR research exposures:

○ Yes ● No

15.3 * This study may generate genetic data that may be broadly shared (e.g. submitted to NIH for Genome-Wide Association Studies (GWAS) in dbGaP, TCGA, etc):

○ Yes ● No

15.4 * This study involves administration of vaccines produced using recombinant DNA technologies to human subjects:

○ Yes ● No

15.5 * This study involves human gene transfer (NOTE: Requires NIH Recombinant DNA Advisory Committee (RAC) review prior to CHR approval):

○ Yes ● No

15.6 This study involves other regulated materials and requires approval and/or authorization from the following regulatory committees:

☐ Institutional Biological Safety Committee (IBC)

Specify BUA #:

☐ Institutional Animal Care and Use Committee (IACUC)

Specify IACUC #:

☐ Radiation Safety Committee

Specify RUA #:

☐ Radioactive Drug Research Committee (RDRC)

Specify RDRC #:

☐ Controlled Substances

16.0 Procedures

16.1 * Procedures/Methods (Help Text updated 9/13) For clinical research list all study procedures, test and treatments required for this study, including when and how often they will be performed. If there are no clinical procedures, describe the Methods:

All patients in ZSFG Peds Urgent Care during study hours meeting inclusion criteria will be asked by a designated study research assistant accompanied by a study designated trained SFGH Health Advocate Research Assistant (HARA) if they are interested in the study. (See Introduction Script attached.) If there is time in the urgent care private patient room (where there are often long waiting periods for providers), the research assistant will review consents and HIPAA authorization forms with family. Otherwise, following the clinic visit, the study research assistant will escort interested patient families to the ZSFG Health Advocate office on ZSFG 6A (on the same floor and down the hall from the urgent care clinic) for eligibility review and consent process. The study research assistant will assign eligible participants a study ID#, which will be stored in a password protected REDCap file on the study's encrypted and password-protected laptop.

The Research Assistant will review consent with eligible caregivers and patients old enough to participate in consent process. Consent will include documenting study ID, completing caregiver name and contact information and child MR#. (NB: Any duplicate MRN will not be eligible for participation.) One consent form will be used for participants randomized into the intervention arm or the control arm of the study (See attached).

Consenting caregivers will be given a copy of the study consent forms. They will also be asked to review and sign a HIPAA authorization form to enable access to separate ZSFG HA surveys/follow up and other health care records. In addition to caregiver consent, children 7 and older also will complete assent forms. (See attached study assent forms.) With caregiver permission, children will be offered entertainment activities including bubbles and age appropriate books, games, and videos. Before caregivers complete health surveys with the Research Assistant and HARA, children 2 yo and over and all caregivers will be given a cup of water and asked to swoosh mouths with water and then swallow water. This needs to be completed 20 minutes before saliva and buccal mucosal cell collection. Following water swoosh, all participating caregivers will complete a baseline survey conducted by Research Assistant and HARA on REDCap using a computer tablet. (See attached documents for baseline and follow up surveys.) There is one self-completion portion of the survey, which includes the Patient Health Questionnaire (PHQ-8) and measures for social support and stress, that participants can read and complete themselves or ask for assistance on. After completing the survey, all participating caregivers and children then will have hair cortisol and oral buccal mucosal cell and saliva samples collected. Participants will then be randomized into the intervention arm or the control arm of the study.

Randomizaton will occur at the participant level using a stratified randomization method in the computer-based program REDCap. This stratified method will be used to ensure a balance of Spanish speaking families and English speaking families within the groups. After completing the study survey and biomarkers collection, the Research Assistant will leave participant dyads wth the HARA for randomization. The HARA will randomly assign participants to either the intervention or the control group. The Research Assistant will have no knowledge of or access to participants' group assignments.

If a family is randomized into the intervention group, the HARA will have access to the social needs screening results obatined through the health survey. The HARA will proceed with the health advocacy intervention follwing the procedures of the SFGH Health Advocacy program and attempt to address unmet needs identified by the caregiver in the survey. This involves working directly with these participants to identify and address any social needs the family may have such as access to food, living conditions and financial security. This may also include providing referrals to outside agencies, referral to legal aid within the clinic, help with applications for social benefits, or other services (See attached HARA Initial Action Plan and FollowUp Survey). Participants in the intervention group who are also ZSFG Pediatric Primary Care patients will have receipt of any direct assistance provided by the HARA documented in their child's medical record indicating services provided. All members of the intervention group will receive a written handout containing information about community resources related to the social needs we ask about in the survey.

The HARA will follow-up with members of the intervention group for a maximum of 3 months by telephone to continue to assess progress towards addressing social needs and to provide further help as needed. When relevant, HARA will send blank program forms to families or other publicly available program information via email or U.S. post to members of the intervention group. If a patient does not respond to telephone follow-ups for over 1 month, HARA may also send secure email to initiate follow-up contact.

If participant dyads are randomized into the control group, the HARA will provide the family with a written handout containing information about community resources related to the social needs we ask about in the survey.

Complete scripts for the biomarker specimen collections are found in Appendices A and B. Following surveys, hair, saliva and buccal mucosal cells will be collected non-invasively. These measures will be obtained immediately after enrollment and at the six month follow up timepoint.

Appendix A.

Hair Collection Procedure

The steps involved in collecting a hair sample from either participant when hair is longer than 3cm are as follows:

1. Carefully comb hair and part laterally at the posterior vertex
2. Take the upper layer of hair and retract either using the hair clip(s), hair pin(s), or having the subject hold their hair above the part.
3. Create a thinned layer of hair (1-2 hairs thick) parallel to the floor using the tail of a parting comb to weave back and forth through a layer of hair. Hold this between your fingers.
4. Slide fingers distally along the hair length; fit the shears up against the scalp across a 4-5 cm length. Cut across this length.
5. Hold all the hair firmly until the cut is complete slowly pulling the cut strands from the area below the part. You should obtain more than 50 strands in this manner. Ideally one should obtain at a minimum 50 single hair stands at least 3 cm in length.
6. Slowly and carefully bring the hair samples together while keeping track of the scalp end and place on the foil.
7. Carefully tape the hair to the foil using painter’s masking tape on the distal ends. Mark the scalp end on the foil with the permanent marker. The foil is carefully folded and closed and labeled with subject enrollment ID.

The steps involved in taking a hair sample from either participant when hair is shorter than 3cm are as follows:

1. Carefully comb hair and part laterally at the posterior vertex.
2. Take the upper layer of hair and retract having the subject hold their hair above the part. Create a thin layer of hair (1-2 hairs thick) parallel to the floor using the open thinning shears.
3. Position the hair below the part into the envelope such that the hair along the part is falling into the envelope when trimmed by the thinning shears.
4. Cut the hair that is overlapping into the envelope with the thinning shears, as close to the scalp as possible, while allowing the trimmed hair to fall into the envelope. The total length of this cut is a function of how short the subject’s hair might be. Generally a lateral cut of 6-8 cm will produce adequate hair for assay. The present method requires at least 8-10 mg of hair to process.
5. Take the hair that was caught by the envelope and transfer it to the aluminum foil for storage. The foil is carefully folded and closed and labeled with subject ID. Due to the short length of these samples the entire length will be processed after determining the average hair length in cm.

Appendix B

Saliva and Buccal Mucosal Cell Sample Collection Procedure:

As with the hair sample collection, the research assistant will collect the mother's saliva and buccal cell sample while the child is with his/her caregiver. Then the research assistant will collect the child's sample. The steps involved in collected samples of saliva and buccal cells are as follows:

1. Make sure the child or adult has no food or gum in his/her mouth.
2. Put on non-latex gloves
3. Pull open the package from one end
4. Remove one of the swabs from the tube
5. Ask child or mother to open his/her mouth; use a tongue depressor if necessary.
6. Insert the swab into the mouth and rub firmly against the inside of the cheek and/or underneath lower and upper lips.
7. Continue rubbing in broad strokes for approximately 30 seconds to 1 minute. For maximum DNA collection, expand the sampling area to both sides of the mouth and use both sides of swab.
8. Remove the swab from the mouth, slide the plastic cap over the swab handle, with the flat side of the cap facing upwards and the swab facing downwards.
9. Insert the swab into the clear plastic tube and push the cap in place.
10. Hold the cap firmly on the tube while pulling the swab handle outwards to release the swab material into the tube.
11. Close the cap; the tube is now completely sealed.
12. Insert saliva swab into mouth until saturated.
13. Remove swab and insert into plastic tube; close cap; the tube is now completely sealed.

After collection of biomarker hair and saliva/buccal mucosal cell samples, family will receive the first $50 gift card for participation. At five months post-enrollment, research assistant will contact family by phone or email to arrange 6 month follow up visit (may occur no earlier than 5 months and no later than 9 months post enrollment). Participants will be contacted up to 5 times by phone and/or email to arrange in-person follow-up for repeat survey and biomarker collection activities.

At the six month visit, child and caregivers will meet with the Research Assistant in the same private room on 6A, an interview room located in the Clinical Research Services (CRS) facilitates located at SFGH hospital one floor below pediatric urgent care in unit 5B, or in another community location that ensures participants' confidentiality where surveys will be conducted and non-invasive biomarker specimens collected from caregiver and child (repeat of collection procedures as above.)

Families will be offered parking stickers to the 23rd Street parking garage at ZSFG or MUNI passes to assist with transportation for the follow-up visit.

At end of follow up visit, family will receive second gift card of $50 for the family's study participation. All participating families randomized into the control arm of the study will be offered health advocacy services after completing this 6 month follow-up visit.

Caregivers placed in the intervention arm of the study who have completed the follow-up visit will be invited to participate in a key informant interview with a Research Assistant about their experiences working with the health advocate patient navigator and accessing services. Caregivers will be invited to participate in the interview directly after completing their 6-month follow-up interview or by telephone at a later date. Interviews Caregivers will receive up to 3 phone calls inviting them to participate in this follow-up interview. Participants who agree to participate in this follow-up interview will sign a separate consent document explained to them by a Research Assistant. Participants who complete the key informant interview will receive a $25.00 VISA gift card.

Child health records will be reviewed 12 months following participant enrollment date.

If you have a procedure table, attach it to the submission with your other study documents.

16.2 Interviews, questionnaires, and/or surveys will be administered or focus groups will be conducted:

(•) Yes ( ) No

List any standard instruments used for this study:

Patients eligible for enrollment, who consent to be in the study, will be subject to:

a. Social, physical and mental health status and health care utilization survey conducted via laptop using REDCap. (See attached baseline and follow up surveys.) Survey is administered in person by fully

bilingual English/Spanish research assistants. There is a short section with depression, social support and stress measures typically collected by self-report at the end of both baseline and follow up survey where patients can self-complete while research assistant is available for assistance.
b. Follow-up surveys will be conducted at 6 months post study enrollment, again by bilingual English /Spanish research assistants. (See attached follow up survey.) Research Assistant will call or email a maximum of 3 times each to arrange or conduct phone follow-up. If there is no response to the phone calls and email, patients will receive a letter addressed to the address of record asking them to phone the research line if they are willing to complete the study survey. Each survey is anticipated to last a maximum of 20 minutes. After each survey administration and biomarker collection, participating families will receive $50 VISA gift card.

For patients who are unable to attend a follow-up visit in-person, but are still willing to participate in the study, the Research Assistant will offer to complete the survey by phone or provide patients with an online link to the survey. Patients who complete only the survey and do not come in for biomarker collection will receive a $25 VISA gift card.

Caregivers who consent to participate in the follow-up key informant interview will complete a 60-minute in-person qualitative interview with a Research Assistant about their experiences working with the health advocate patient navigator and accessing services. Participants who complete the key informant interview will receive a $25.00 VISA gift card.

All Research Assistants will complete CITI and HIPAA training in addition to receiving training in research /consent rules and regulations, cultural sensitivity, legal and social needs screening and intervention, as well as survey administration techniques.

Attachments
Research study introductory script (for clinic staff)
Baseline survey (at enrollment)
Follow up survey (6 months)
Consent documents, including HIPAA authorizations and child assent forms
Key informant qualitative interview guide
Key informant telephone consent script
Key informant qualitative interview consent document

Attach any non-standard instruments at the end of the application.

16.3 Conduct of study procedures or tests off-site by non-UCSF personnel:

○ Yes ◉ No

If yes, explain:

16.4 Sharing of experimental research test results with subjects or their care providers:

○ Yes ◉ No

If yes, explain:

16.5 * Specimen collection for future research and/or specimen repository/bank administration:

◉ Yes ○ No

16.6 Time commitment (per visit and in total):

Initial Visit @ Enrollment:
Consent Process: approx 30 minutes
Baseline survey: approx 15-20 minutes
Biomarker collection (child and caregiver): 10 minutes

Follow up Visit at 6 months:
Follow up survey: approx 15 minutes
Biomarker collection (child and caregiver): 10 minutes

Follow up Key Informant interview post 6 month follow-up:
Consent Process: approx 15 minutes
Interview: 60 minutes

16.7 Locations:

Study activities will take place in the urgent care clinic and/or in the ZSFG Health Advocates program office two offices down from Children's Health Center. Follow-up visits may also be conducted in the interview rooms located in the Clinical Research Services (CRS) facilitates located one floor below pediatric urgent care in unit 5B. At the patient's request, follow up surveys and biomarker collection can take place in the patient's home.

In the event that the research assistant is asked to collect data in subjects' homes the following safety procedures will be in place: 1) key study personnel will be aware of the scheduled appointment and be available by phone during the home visit, 2) the research assistant will carry a study cell phone and 3) the research assistant will receive basic home visitor safety training including awareness of surroundings, neighborhood safety and how to communicate appropriate boundaries.

Analysis and Interpretation of Biological Data. Biological specimens collected as part of this research will be analyzed and stored at Dr. Michael Meaney's lab at the Research Center of the Douglas Mental Health University Institute, McGill University in Montreal, Canada. De-identified patient-level survey data collected during the course of the study will be transferred securely to Dr. Michael Meaney's lab at the Research Center of the Douglas Mental Health University Institute, McGill University for use in the interpretation of the biological data analyses. Transfer of patient-level survey data and biological samples are clearly stated in a data sharing agreement with the Research Center of the Douglas Mental Health University Institute, McGill University.

16.8 Describe the resources in place to conduct this study in a way that assures protection of the rights and welfare of participants:

1. All study participants are required to review and sign informed consent prior to participation and asked to complete HIPAA consents.
2. All data are stored on secure servers.
3. All staff required to complete HIPAA and CITI training, in addition to legal, cultural sensitivity training.
4. A limited number of study staff will have access to study data.
5. PHI will be linked by study ID to main data files and only accessed as needed by research assistants with clearance to view pertinent files.
6. All biomarkers will be sent to analyzing labs with study enrollment IDs and no additional patient identifiers.

17.0 Specimen Collection for Future Research and/or Specimen Repository/Bank Administration

17.1 Specimens are (check all that apply):

- [ ] Surplus clinical specimens from a diagnostic or therapeutic procedure
- [x] Specimens collected for research purposes only
- [ ] Other

If Other, explain:

17.2 Types of specimens:

- [ ] Blood

☐ Tissue (describe below):
☐ Existing/archival materials (name source below): --
☑ Other (describe below):

Describe and/or name source:

Oral buccal epithelial cells swabs and saliva samples will be collected from children and caregivers. These procedures are described in the procedures section of the application. The samples will be stored for analysis of inflammatory cytokines and DNA methylation in Michael Meaney's lab in Montreal. Dr. Meaney is expected to conduct these analyses and possibly other analyses of biomarkers on these samples when funding is available. Sample storage is explained carefully in the consent documents.

17.3 Consent will be obtained via:

☐ Separate specimen banking consent form
☑ Specimen banking section within a main research study consent form
☐ Surgical consent form with tissue donation brochure

17.4 Specimens will ultimately be stored (check all that apply):

UCSF

☐ UCSF repository/bank being established under this protocol
☐ Existing UCSF specimen repository/bank with CHR approval

Provide the name of the bank and CHR approval number (if not being banked at UCSF under this protocol):

Outside Entity

☐ Cooperative group bank
☐ NIH
☐ Other university
☐ Industry sponsor
☑ Other

Specify to what institution, cooperative group or company specimens will be transferred:

Michael Meaney's Lab
Research Center of the Douglas Mental Health University Institute/McGill University
6875 LaSalle Boulevard
Montréal (Québec) H4H 1R3
Canada

17.5 Direct identifiers will be sent with specimens or shared with other researchers and/or outside entities:

○ Yes
◉ No
○ N/A - Specimens will not be shared with others

If **Yes**, which identifiers will be sent with specimens:

☐ Name
☐ Date of birth
☐ Social Security number
☐ Medical record number

☐ Address
☐ Phone number
☐ Email address
☐ Other dates (surgery date, clinic visit dates, etc.)

If **Yes**, provide a justification for sending direct identifiers with the specimens:

18.0 Alternatives

18.1 Study drug or treatment is available off-study:

○ Yes
○ No
◉ Not applicable

18.2 * Is there a standard of care (SOC) or usual care that would be offered to prospective subjects at UCSF (or the study site) if they did not participate:

○ Yes ◉ No

If yes, describe the SOC or usual care that patients would receive if they choose not to participate:

18.3 Describe other alternatives to study participation that are available to prospective subjects:

N/A

19.0 Risks and Benefits

19.1 * Risks and discomforts:

There are no physical risks to the participants from taking part in the treatment protocol part of the study. There is a risk that the questions asked during the assessments or the issues that are brought up in treatment may be anxiety-provoking or upsetting, but participants will be told that they have the right to refuse to answer any questions or to withdraw from participation at any time.
Participation in the biological samples sub-study poses minimal risks to participants. They are as follows and are listed on the consent form:
Hair Sample: There is no risk involved in taking a hair sample. About 50-100 strands of hair will be taken from the back of the mother's and child's head, using thinning shears. Thinning shears are special scissors used by hairdressers and barbers to reduce hair thickness, to create special texturizing effects, or to blend layered hair—they remove hair without making it look like hair is missing. For some hair types, this thinning technique will be accomplished with hair styling shears. The biological samples (hair and oral swabs) will be collected from caregiver and child by a trained research assistant in a private room. Before the sample collection, the research assistant will demonstrate the sample collection with a short video clip. To give you an idea of how much 50 strands of hair looks like, humans typically lose 50-150 hairs per day.
Cheek Cell Sample: There is no risk involved in collecting a sample of cheek cells other than minor, temporary irritation that may result from rubbing the swab against the inside of the cheek.
Analyzing biological samples: Information on maternal and child's genes, stress hormones, immune system activity, and cellular aging will be acquired through the biological samples. Donating biological material could result in a loss of confidentiality. We will make every effort to preserve the confidentiality of this child's biological information, just as we do for all health information. After samples are collected we remove all identifying information, such as names and addresses, and analyses of study data will be conducted without any access to participants' identifying information

19.2 Steps taken to minimize risks to subjects:

All participants are advised that they have the right to decline to answer any questions that may make them feel uncomfortable.
Participation in the study is optional. There are no repercussions for non-participation. Children and caregivers consenting for the study and those who do not will receive the same clinical treatment.
Participants in the study can refuse future participation at any time during the course of treatment; their samples will be removed from analyses.
Both mother and child will give their hair, saliva and buccal cell samples together in the private office with the research assistant present. The child will give his/her samples while sitting in mother's lap or in his/her own chair. At no time will the child or mother be left alone.
As it is explained to participants, collection of biological samples will help us understand how the Health Advocates program may impact physical health.

19.3 Benefits to subjects:

○ Yes ◉ No

If yes, describe:

19.4 Benefits to society:

If our hypotheses are correct, then connecting patients with health advocate navigators will decrease social needs, decrease caregivers' missed work days, and improve child school attendance, decrease hair cortisol and salivary/buccal mucosal cell inflammatory markers, and decrease avoidable health care utilization.

19.5 Explain why the risks to subjects are reasonable:

We feel the benefits of this study far outweigh the minimal risks. With information from the study, we will be able support program sustainability by better understanding how the program impacts social determinants of health.

20.0 Confidentiality and Privacy

20.1 Plans for maintaining privacy in the research setting:

The consent form will stipulate that information provided by the subjects will remain strictly confidential, with access limited to the research staff. All collected data will be stored in the secure, UCSF HIPAA secure REDCap system. The HIPAA data will be collected in a separate REDCap file linked via Study Enrollment Number to the completed survey files. Access to the REDCap system will be through the use of log-ins and passwords. Publications or presentation of findings will not include information identifying the subjects. Biomarker samples will be identified only by study enrollment number, so any information stored outside REDCap (other than consents) will not have identifying information other than study enrollment number. Signed consent forms will also be in a locked filing cabinet in a locked office in the SFGH Peds Primary Care Clinic/Urgent Care and study research offices..

Data collected from REDCap will be accessed through a secure website. Once all survey data are collected and other EHR data are requested, all data will be transferred to MyResearch files.
**REDCap is hosted at the UCSF Minnesota Street data center and is housed in a locked and guarded data center staffed at all hours (24 X 7). Entrance to the data center requires use of a card key to unlock the data center door and a second card key lock secures the cage that the servers reside within. The security of the data center is further protected by an Operations desk that is staffed 24x7 and by a security camera system. REDCap servers are guarded by multiple firewall and intrusion detection systems. All electronic connections to the REDCap environment are encrypted. The REDCap production system is comprised of a web server front-end and a MySQL database server back-end. The web server resides in a demilitarized zone to ensure that survey participants are able to access REDCap surveys from any device connected to the Internet. The MySQL server back-end resides in the protected ISU subnet that is protected by UCSF maintained firewalls. The data stored in the REDCap MySQL database server can be accessed by the REDCap end users by logging into https://redcap.ucsf.edu and opening the REDCap project(s) that they have been granted access to by the owners of the projects. Only ITS and ISU system administrators are authorized to access the back-end database server directly by

logging into the virtual private network for the database server resides in. REDCap databases are backed up on a regularly scheduled basis. The backup data files are kept in a secured environment and are available for recovery.

**MyResearch is a secure data hosting service for researchers. This service provides research teams with a professionally managed, secure, web-based, collaborative environment in which to manage files containing sensitive data. MyResearch also provides remote desktop capability with application and database services that allow investigators to view, manipulate, and save their data entirely in a protected environment without requiring files to be stored on their own computers. Applications such as SAS or Excel run on the MyResearch servers in a UCSF secure data center but they appear on the user's own screen as if they were running locally on the user's computer.

20.2 Possible consequences to subjects resulting from a loss of privacy:

In addition to biomarkers data, caregivers' household information on social and environmental needs, health and health care utilization are all included in the final study dataset. The survey data will be linked via study enrollment number to the PHI data files. A privacy breech that includes this information is unlikely to have any negative consequences.

20.3 Study data are:

- [ ] Derived from the Integrated Data Repository (IDR) or The Health Record Data Service (THREDS) at SFGH
- [x] Derived from a medical record (e.g. APeX, OnCore, etc. Identify source below)
- [x] Added to the hospital or clinical medical record
- [x] Created or collected as part of health care
- [ ] Used to make health care decisions
- [x] Obtained from the subject, including interviews, questionnaires
- [ ] Obtained from a foreign country or countries only
- [ ] Obtained from records open to the public
- [ ] Obtained from existing research records
- [ ] None of the above

If **derived from a medical record**, identify source:

SFGH LCR and eCW EHR data. We will associate EHR data and study survey data.

20.4 Identifiers may be included in research records:

(●) Yes ( ) No

If **yes**, check all the identifiers that may be included:

- [x] Names
- [x] Dates
- [x] Postal addresses
- [x] Phone numbers
- [ ] Fax numbers
- [x] Email addresses
- [ ] Social Security Numbers*
- [x] Medical record numbers
- [ ] Health plan numbers
- [ ] Account numbers
- [ ] License or certificate numbers
- [ ] Vehicle ID numbers
- [ ] Device identifiers or serial numbers
- [ ] Web URLs

- [ ] IP address numbers
- [ ] Biometric identifiers
- [ ] Facial photos or other identifiable images
- [ ] Any other unique identifier

* Required for studies conducted at the VAMC

20.5 Identifiable information might be disclosed as part of study activities:

( ) Yes (•) No

If **yes**, indicate to whom identifiable information may be disclosed:

- [ ] The subject's medical record
- [ ] The study sponsor
- [ ] Collaborators
- [ ] The US Food & Drug Administration (FDA)
- [ ] Others (specify below)
- [ ] A Foreign Country or Countries (specify below)

If **Others**, specify:

20.6 Indicate how data are kept secure and protected from improper use and disclosure (check all that apply): NOTE: Whenever possible, do not store subject identifiers on laptops, PDAs, or other portable devices. If you collect subject identifiers on portable devices, you MUST encrypt the devices.

- [x] Data are stored securely in My Research
- [x] Data are coded; data key is destroyed at end of study
- [x] Data are coded; data key is kept separately and securely
- [x] Data are kept in a locked file cabinet
- [x] Data are kept in a locked office or suite
- [x] Electronic data are protected with a password
- [x] Data are stored on a secure network
- [x] Data are collected/stored using REDCap or REDCap Survey
- [ ] Data are securely stored in OnCore

20.7 Additional measures to assure confidentiality and protect identifiers from improper use and disclosure, if any:

PHI on pediatric patients obtained from electronic health records and from ZSFG HA surveys (not study-related) will be kept on an encrypted, password-protected laptop with password-protected files in MyResearch. Data analyses will be based on data from research surveys and health records data and also kept in MyResearch files. Biomarker data will be sent only accompanied by Study Enrollment number without other identifiers.

20.8 This study may collect information that State or Federal law requires to be reported to other officials or ethically requires action:

(•) Yes ( ) No

Explain:

While we do not directly ask the suicidality question from the PHQ-9, any cases where the participants report suicidality the research assistant will immediately notify the attending physician on duty in the Pediatric Urgent Care Center.

20.9 This study will be issued a Certificate of Confidentiality:

○ Yes ◉ No

21.0 Subjects

21.1 Check all types of subjects that may be enrolled:

- [ ] Inpatients
- [x] Outpatients
- [ ] Healthy volunteers
- [ ] Staff of UCSF or affiliated institutions

21.2 Additional vulnerable populations:

- [x] Children
- [ ] Subjects unable to consent for themselves
- [ ] Subjects unable to consent for themselves (emergency setting)
- [ ] Subjects with diminished capacity to consent
- [x] Subjects unable to read, speak or understand English
- [ ] Pregnant women
- [ ] Fetuses
- [ ] Neonates
- [ ] Prisoners
- [x] Economically or educationally disadvantaged persons
- [ ] Investigators’ staff
- [ ] Students

Explain why it is appropriate to include the types of subjects checked above in this particular study:

We are conducting this study with caregivers of patients in the ZSFG Peds Urgent Care Clinic, where many caregivers are unable to read/write English, and many are socioeconomically disadvantaged. Since the aim of the study is to document the impact of better connect patients who need resources with available community services, it is essential to recruit these families. The study is aimed at improving pediatric health outcomes so recruiting from pediatric populations is an additional requirement. We have checked the "children" box because we are accessing medical records of the index child (child receiving care at ZSFG on day of enrollment) at 12 months following enrollment.

Describe the additional safeguards that have been included in the study to protect the rights and welfare of these subjects and minimize coercion or undue influence:

We are consenting caregivers who are parents or legal guardians who have knowledge of the pediatric patients' living environment. This should minimize risks to the rights and welfare of pediatric patients. We are adding an adolescent assent requirement for using EMR utilization data at 12 months following household enrollment in the study. EMR data will be linked with study survey and biomarker data. We will obtain HIPAA authorization from all participants for whom these links will be made.

22.0 Inclusion of Children in Research

22.1 This study will enroll children who can legally consent for themselves:

○ Yes ◉ No

If **yes,** explain why they can consent for themselves in the research setting:

If you will **ONLY** be enrolling children who can legally consent for themselves, press **SAVE and CONTINUE** to skip the rest of this section.

22.2 Select all the regulatory categories that apply:

- [x] No greater than minimal risk (45 CFR 46.404, 21 CFR 50.51)
- [ ] Greater than minimal risk but presenting prospect of direct benefit (45 CFR 46.405, 21 CFR 50.52)
- [ ] Greater than minimal risk (though only a minor increase over minimal risk) and no prospect of direct benefit but likely to yield generalizable knowledge about the subjects disorder or condition (45 CFR 46.406, 21 CFR 50.53)
- [ ] Research not otherwise approvable which presents an opportunity to understand, prevent, or alleviate a serious problem affecting the health or welfare of children (45 CFR 46.407, 21 CFR 50.54)

Explain why the research in this study falls under the above category or categories:

22.3 Parental permission or waiver:

- [x] Parental permission will be obtained
- [ ] Waiver of parental permission is requested: Parental permission is not a reasonable requirement
- [ ] Waiver of parental permission is requested: The waiver meets the provisions for a waiver of consent set forth in 45 CFR 46.116, Subpart A

If you are requesting a **waiver of parental permission**, explain why the study meets the regulatory criteria for this waiver:

22.4 Assent of children or waiver:

- [x] Assent of children old enough to provide assent will be obtained
- [ ] Waiver of assent is requested: Children cannot be consulted or the research has prospect of direct benefit only available in the study
- [ ] Waiver of assent is requested: The waiver meets the provisions for a waiver of consent set forth in 45 CFR 46.116, Subpart A

If you are requesting a **waiver of child's assent**, explain why the study meets the regulatory criteria for this waiver:

22.5 Documentation of permission and assent (select all that will be used):

- [x] Permission form addressed to the parents
- [x] Simplified assent form addressed to the child, 7-12 years old (parents get separate form)
- [x] Assent form addressed to the child, 13 years and older (for subjects and parents)
- [ ] Assent form addressed to the child, 13 years and older (parents get separate form)

Check one:

- ○ One parent's signature will be obtained
- ○ Two parents' signatures will be obtained

If this study is approvable under .404 or .405 and you plan to get permission from only one parent, explain why you think one parent's permission is sufficient:

The study poses minimal risk to children and caregivers.

22.6 This study may enroll wards of the state:

○ Yes ◉ No

23.0 Inclusion of Non-English Speaking Subjects

23.1 Indicate which method(s) you will use to consent non-English speaking subjects:

- [x] Preferred Method—Consent form and other study documents will be available in the subject's primary language Personnel able to discuss participation in the patient's language will be present for the consent process.
- [ ] Short-Form—A qualified interpreter will translate the consent form verbally, and subjects will be given the Experimental Subject's Bill of Rights in their primary language, following instructions in Those Who do not Read, Speak or Understand English for required witnessing and signatures

23.2 Explain how you will maintain the ability to communicate with non-English speakers throughout their participation in the study:

We plan to have a fully bilingual, bicultural trained research assistant available during study hours to communicate with Spanish-speaking participants. The consent document will be available in English and in Spanish. Interpreter services will not be used to consent patients for this study.

24.0 Recruitment

24.1 * Methods (check all that apply):

- [x] Study investigators (and/or affiliated nurses or staff) recruit their own patients directly in person or by phone.
- [ ] Study investigators recruit their own patients by letter. Attach the letter for review.
- [ ] Study investigators send a "Dear Doctor" letter to colleagues asking for referrals of eligible patients. If interested, the patient will contact the PI or the PI may directly recruit the patients (with documented permission from the patient). Investigators may give the referring physicians a study information sheet for the patients.
- [ ] Study investigators provide their colleagues with a "Dear Patient" letter describing the study. This letter can be signed by the treating physicians and would inform the patients how to contact the study investigators. The study investigators may not have access to patient names and addresses for mailing
- [ ] Advertisements, notices, and/or media used to recruit subjects. Interested subjects initiate contact with study investigators. Attach ads, notices, or media text for review. In section below, please explain where ads will be posted.
- [ ] Study investigators identify prospective subjects through chart review. (Study investigators request a Waiver of Authorization for recruitment purposes.)
- [ ] Large-scale epidemiological studies and/or population-based studies: Prospective subjects are identified through a registry or medical records and contacted by someone other than their personal physician. (Study investigators request a Waiver of Authorization for recruitment purposes.)
- [ ] Direct contact of potential subjects who have previously given consent to be contacted for participation in research. Clinic or program develops a CHR-approved recruitment protocol that asks patients if they agree to be contacted for research (a recruitment database) or consent for future contact was documented using the consent form for another CHR-approved study.
- [ ] Study investigators list the study on the School of Medicine list of UCSF Clinical Trials website or a similarly managed site. Interested subjects initiate contact with investigators.
- [ ] Study investigators recruit potential subjects who are unknown to them through methods such as snowball sampling, direct approach, use of social networks, and random digit dialing.
- [ ] Other

If **Other**, explain:

24.2 * How, when, and by whom eligibility will be determined:

A designated research study assistant located in pediatric urgent care will introduce study and determine initial eligibility based on language and patient age. Once family has expressed interest in the study, the research assistant will ensure family members meet all eligibility requirements by asking questions related to inclusion/exclusion criteria.

24.3 * How, when, where and by whom potential subjects will be approached:

Caregivers will be approached in the urgent care patient clinic room or waiting room by the study research assistant and a study designated trained ZSFG Health Advocate research assistant and asked whether they are interested in talking about participating in a study on the effectiveness of the ZSFG hospital Health Advocate program. If so, patients will be screened for eligibility. In clinic rooms, while waiting for the provider, the research assistant will consent eligible families and complete study activities with the assistance of the study designated ZSFG Health Advocate research assisant. If the clinic visit ends before study activities are completed, the research assistant and the designated ZSFG Health Advocate research assistant will walk the family down the hall to the research office where the remaining study activities will occur. All study activities will be deferred for provider-patient interactions. This is how we ran our intial study in the Peds Primary and Urgent Care Clinics and this was well-received by clinic staff.

24.4 * Protected health information (PHI) will be accessed prior to obtaining consent:

○ Yes ◉ No

25.0 Waiver of Consent/Authorization for Recruitment Purposes

This section is required when study investigators (and/or affiliated nurses or staff) recruit their own patients directly.

25.1 * Study personnel need to access protected health information (PHI) during the recruitment process and it is not practicable to obtain informed consent until potential subjects have been identified:

◉ Yes

If **no**, a waiver of consent/authorization is NOT needed.

25.2 * A waiver for screening of health records to identify potential subjects poses no more than minimal risk to privacy for participants:

◉ Yes

If **no**, a waiver of authorization can NOT be granted.

25.3 * Screening health records prior to obtaining consent will not adversely affect subjects' rights and welfare:

◉ Yes

If **no**, a waiver of authorization can NOT be granted.

25.4 * Check all the identifiers that will be collected prior to obtaining informed consent:

- [x] Names
- [ ] Dates
- [ ] Postal addresses

- [ ] Phone numbers
- [ ] Fax numbers
- [ ] Email addresses
- [ ] Social Security Numbers*
- [ ] Medical record numbers
- [ ] Health plan numbers
- [ ] Account numbers
- [ ] License or certificate numbers
- [ ] Vehicle ID numbers
- [ ] Device identifiers or serial numbers
- [ ] Web URLs
- [ ] IP address numbers
- [ ] Biometric identifiers
- [ ] Facial photos or other identifiable images
- [ ] Any other unique identifier
- [ ] None

Note: HIPAA rules require that you collect the minimum necessary.

25.5 * Describe any health information that will be collected prior to obtaining informed consent:

No records will actually be collected by study personnel before informed consents are obtained. Study staff will ask re: parent/caregiver willingness to participate in study activities.

Note: HIPAA requires that you collect the minimum necessary.

25.6 * Describe your plan to destroy the identifiers at the earliest opportunity consistent with the research or provide a health or research justification for retaining the identifiers, or indicate and explain that retention is required by law:

Identifiers will be destroyed at study end by erasing all electronic linking files.

26.0 Informed Consent

26.1 * Methods (check all that apply):

- [x] Signed consent will be obtained from subjects and/or parents (if subjects are minors)
- [ ] Verbal consent will be obtained from subjects using an information sheet or script
- [ ] Electronic consent will be obtained from subjects via the web or email
- [ ] Implied consent will be obtained via mail, the web or email
- [ ] Signed consent will be obtained from surrogates
- [ ] Emergency waiver of consent is being requested for subjects unable to provide consent
- [ ] Informed consent will not be obtained

26.2 * Process for obtaining informed consent:

a) The consent will take place in ZSFG Children's Health Center Urgent Care Clinic.
b) Families will be able to ask questions of the fully bilingual/bicultural Eng/Spanish research assistants before signing consents. They will be able to participate in the study or decline and their decision will not affect the quality of care that they receive.
c) We will have written consents, assent forms for child participants, and HIPAA authorizations in both English and Spanish.

26.3 * How investigators will make sure subjects understand the information provided to them:

We will have bilingual research assistants available to answer any questions.

27.0 Financial Considerations

27.1 Subjects payment or compensation method (check all that apply):

Payments will be (check all that apply):

- [ ] Subjects will not be paid
- [ ] Cash
- [ ] Check
- [ ] Debit card
- [x] Gift card
- [ ] Reimbursement for parking and other expenses
- [x] Other:

Specify **Other**:

Families will be offered parking stickers to the 23rd Street parking garage at ZSFG or MUNI passes to assist with transportation for the follow-up visit.

27.2 Describe the schedule and amounts of payments, including the total subjects can receive for completing the study. If deviating from recommendations in Subject Payment Guidelines, include specific justification below.

Each participating dyad (caregiver + child) will be paid up to $100 in gift cards for taking part in this study. Each family will receive a $50 gift card after each of the two visits.

In instances where families do not return for an in-person follow-up visit and complete only the survey by telephone or online, they will receive a $25 gift card for the follow-up visit. In these cases, the participating dyad will be paid a total of $75 for taking part in the study.

Caregivers who complete the key informant interview will receive an additional $25 gift card at completion of the in-person interview.

27.3 Costs to Subjects: Will subjects or their insurance be charged for any study procedures?

○ Yes ◉ No

If **yes,** describe those costs below, and compare subjects' costs to the costs associated with alternative care off-study. Finally, explain why it is appropriate to charge those costs to the subjects.

28.0 CTSI Screening Questions

28.1 * This study will be carried out at one of the UCSF Clinical Research Services (CRS) centers or will utilize CRS services. CRS centers are at the following sites:

- **SFGH Clinical Research Center**
- **Moffitt Adult Clinical Research Center**
- **Moffitt Hospital Pediatrics & NCRC**
- **Mount Zion Hospital Clinical Research Center**
- **Tenderloin Center**
- **CHORI Children's Hospital Pediatrics & Adult Clinical Research Center**
- **Kaiser Oakland Research Unit**
- **SF VA Medical Center Clinical Research Unit**

Please note: Effective 3/1/14, the CRS form will no longer be completed and submitted in iRIS. The CRS budget request form can be found at: https://accelerate.ucsf.edu/files/crs

/BudgetRequest2015.docx. **Follow the instructions on the form to submit. Even if you click 'Yes' to this question, the form will no longer proceed to the Clinical Research Services (CRS) Application Form section.**

(•) Yes ( ) No

28.2 This project involves community-based research:

( ) Yes (•) No

28.3 This project involves practice-based research:

( ) Yes (•) No

29.0 End of Study Application

29.1 **End of Study Application Form** **To continue working on the Study Application: Click on the section you need to edit in the left-hand menu. Remember to save through the entire Study Application after making changes. If you are done working on the Study Application: Click Save and Continue. If this is a new study, you will automatically enter the Initial Review Submission Packet form, where you can attach consent forms or other study documents. Review the Initial Review Submission Checklist for a list of required attachments. Answer all questions and attach all required documents to speed up your approval.**
