# Supplementary material for: Effects of In-Person Assistance vs Personalized Written Resources About Social Services on Household Social Risks and Child and Caregiver Health: A Randomized Clinical Trial
Source: JAMA Netw Open. 2020 Mar 10;3(3):e200701. doi: 10.1001/jamanetworkopen.2020.0701 (PMC7064877; doi:10.1001/jamanetworkopen.2020.0701)
Supplement: Supplement 2. — eAppendix. Social Risk Screening [file jamanetwopen-3-e200701-s002.pdf]

Supplementary Online Content

Gottlieb LM, Adler NE, Wing H, et al. Effects of in-person assistance vs personalized written resources about social services on household social risks and child and caregiver health: a randomized clinical trial. *JAMA Netw Open*. 2020;3(3):e200701. doi:10.1001/jamanetworkopen.2020.0701

eAppendix. Social Risk Screening

This supplementary material has been provided by the authors to give readers additional information about their work.

© 2020 Gottlieb LM et al. *JAMA Network Open.*

eAppendix. Social Risk Screening

I'm going to read you a list of common social issues affecting our families in the ZSFG community.

Please tell me if you are currently concerned about any of the following for you or members of your household…
(Answers are YES/NO)

1. Running out of food before having enough money or food stamps to buy more
2. Unstable housing including eviction, foreclosure, homelessness or staying with friends/family
3. Problems paying bills, like electric, gas, water, or phone bills
4. Housing problems like mold, insects, rats, or mice
5. Difficulty finding a job
6. A disability interfering with the ability to work
7. Problems with a current or former job, like unpaid wages, workers comp, discrimination or harassment
8. Difficulty obtaining unemployment insurance
9. Getting cut off from or denied from programs that provide income support, like Cal Fresh (food stamps), CalWorks, etc
10. Having no health insurance
11. Having no primary care provider for your child or other household member
12. Receiving medical or pharmacy bills that you cannot afford
13. Difficulty finding afterschool activities or opportunities for recreation/education for your child
14. Difficulty finding childcare
15. Bullying
16. Concerns about your or another adult's mental or behavioral health in your household
17. Difficulty affording transportation like MUNI or ADA paratransit
18. Other legal issues not mentioned above, including deportation concerns, child support or family law issues, or violence

© 2020 Gottlieb LM et al. *JAMA Network Open.*
