# Supplementary material for: Effects of In-Person Assistance vs Personalized Written Resources About Social Services on Household Social Risks and Child and Caregiver Health: A Randomized Clinical Trial
Source: JAMA Netw Open. 2020 Mar 10;3(3):e200701. doi: 10.1001/jamanetworkopen.2020.0701 (PMC7064877; doi:10.1001/jamanetworkopen.2020.0701)
Supplement: Supplement 3. — Data Sharing Statement [file jamanetwopen-3-e200701-s003.pdf]

Data Sharing Statement

Gottlieb. Effects of In-Person Assistance vs Personalized Written Resources About Social Services on Household Social Risks and Child and Caregiver Health. *JAMA Netw Open*. Published March 10, 2020. 10.1001/jamanetworkopen.2020.0701

Data
Data available: Yes
Data types: Deidentified participant data
How to access data: Request for data : holly.wing@ucsf.edu
When available: With publication

Supporting Documents
Document types: None

Additional Information
Who can access the data: Researchers whose proposed use of the data has been approved
Types of analyses: Specified uses at the discretion of the UCSF research team
Mechanisms of data availability: After approval of proposal and with a signed data use agreement
